# Anti-inflammatory and antioxidative effects of gallic acid on experimental dry eye: in vitro and in vivo studies

**DOI:** 10.1186/s40662-023-00334-5

**Published:** 2023-05-01

**Authors:** Kexin Li, Qianwen Gong, Bin Lu, Kaiyan Huang, Yixuan Tong, Tinashe Emmanuel Mutsvene, Meng Lin, Zhiqiang Xu, Fan Lu, Xingyi Li, Liang Hu

**Affiliations:** 1 National Clinical Research Center for Ocular Diseases, Eye Hospital, Wenzhou Medical University, Wenzhou, 325027 People’s Republic of China; 2 National Engineering Research Center of Ophthalmology and Optometry, Eye Hospital, Wenzhou Medical University, Wenzhou, 325027 People’s Republic of China; 3 State Key Laboratory of Ophthalmology, Optometry and Visual Science, Eye Hospital, Wenzhou Medical University, Wenzhou, 325027 People’s Republic of China; 4 Institute of Biomedical Engineering, School of Ophthalmology and Optometry, Eye Hospital, Wenzhou Medical University, 270 Xueyuan Road, Wenzhou, 325027 People’s Republic of China

**Keywords:** Corneal epithelial cells, Macrophages, Gallic acid, Dry eye, Inflammation, Oxidative stress

## Abstract

**Background:**

To investigate the anti-inflammatory and antioxidative effects of gallic acid (GA) on human corneal epithelial cells (HCECs) and RAW264.7 macrophages as well as its therapeutic effects in an experimental dry eye (EDE) mouse model.

**Methods:**

A cell counting kit-8 (CCK-8) assay was used to test the cytotoxicity of GA. The effect of GA on cell migration was evaluated using a scratch wound healing assay. The anti-inflammatory and antioxidative effects of GA in vitro were tested using a hypertonic model (HCECs) and an inflammatory model (RAW264.7 cells). The in vivo biocompatibility of GA was detected by irritation tests in rabbits, whereas the preventive and therapeutic effect of GA in vivo was evaluated using a mouse model of EDE.

**Results:**

In the range of 0–100 μM, GA showed no cytotoxicity in RAW264.7 cells or HCECs and did not delay the HCECs monolayer wound healing within 24 h. Ocular tolerance to GA in the in vivo irritation test was good after seven days. In terms of antioxidative activity, GA significantly reduced the intracellular reactive oxygen species (ROS) in lipopolysaccharide (LPS) activated RAW264.7 macrophages and HCECs exposed to hyperosmotic stress. Furthermore, after pre-treatment with GA, the expression levels of nuclear factor E2-related factor 2 (Nrf2), heme oxygenase-1 (HO-1), and NADPH quinone oxidoreductase-1 (NQO-1) were significantly upregulated in RAW264.7 macrophages. GA also exhibits excellent anti-inflammatory properties. This is mainly demonstrated by the ability of GA to effectively downregulate the nuclear transcription factor-κB (NF-κB) pathway in LPS-activated RAW264.7 macrophages and to reduce inflammatory factors, such as nitric oxide (NO), interleukin 6 (IL-6), and tumor necrosis factor alpha (TNF-α). In vivo efficacy testing results in a mouse model of EDE showed that GA can effectively prevent and inhibit the apoptosis of corneal epithelial cells (CECs), reduce inflammatory factors in the cornea and conjunctiva as well as protect goblet cells.

**Conclusion:**

In vitro and in vivo results indicate that GA possesses potent anti-inflammatory and antioxidative properties with no apparent cytotoxicity within the range of 0–100 μM. It is a promising eye drop formulation for the effective prevention and treatment of dry eye disease (DED).

**Supplementary Information:**

The online version contains supplementary material available at 10.1186/s40662-023-00334-5.

## Background

Dry eye disease (DED) is a common ocular surface multifactorial disease with a prevalence of up to 75% in some populations [1, 2]. In recent years, the prevalence of dry eye has been increasing due to the popularity of video terminal equipment, air-conditioned environments, and the increase in the number of people wearing contact lenses [2]. Thus, the direct and indirect economic losses caused by dry eye have also increased [2, 3].

Tear hyperosmolarity and a series of inflammatory events are considered to be important factors in the pathogenesis of dry eye [4]. Hyperosmolar stress of DED activates mitogen-activated protein kinase (MAPK), which activates the master regulator, nuclear transcription factor-κB (NF-κB), leading to the production of interleukin 1 (IL-1), interleukin 6 (IL-6), and tumor necrosis factor alpha (TNF-α). This results in the upregulation of matrix metallopeptidase 9 (MMP-9) which is associated with the disruption of the epithelial corneal barrier [4]. Inflammation is initiated when reactive oxygen species (ROS) induced by hyperosmotic stress activates nucleotide-binding oligomerization domain, leucine-rich repeat and pyrin domain-containing 3 (NLRP3) inflammasomes [5]. Macrophages are important antigen-presenting cells (APCs) during dry eye injury, stimulated by hyperosmolarity or proinflammatory cytokines, macrophage activation is involved in initiating ocular surface adaptive immune responses [4, 6–8]. ROS can cause ocular hyperosmolar stress and persistent ocular surface inflammation. Therefore, inhibition of the cellular inflammatory response and removal of excessive intracellular ROS may facilitate dry eye treatment. Artificial tears currently used to treat dry eye can only relieve the symptoms of DED. The use of steroid eye drops for ocular surface inflammation poses a risk of complications such as cataract and glaucoma. Hence, there is an urgent need to develop safe and effective anti-inflammatory and antioxidant eye drops with fewer side effects.

In recent decades, traditional Chinese medicines such as paclitaxel and artemisinin have gained popularity because of their clear efficacy, low toxicity, and affordability [9–14]. Gallic acid (GA) (i.e., 3, 4, 5-trihydroxybenzoic acid) is a natural botanic phenolic compound, widely found in various plants, fruits, and nuts such as apple peels, pineapples, bananas, lemons, and wine [15–17]. It recently received widespread attention due to its powerful anti-inflammatory and antioxidant properties [18]. In fact, GA can be produced in large quantities through biological and chemical synthesis. Owing to its ability to eradicate ROS, GA acts as an excellent antioxidant and is responsible for many other important biological activities such as anti-inflammatory, anti-cancer, anti-bacterial, anti-viral, and anti-mutagenic effects [19–22]. Moreover, in a variety of animal experiments and clinical trials, toxicity studies have shown that GA rarely causes toxicity and side effects [23–26]. In rats with elastase-induced emphysema, GA has been shown to suppress inflammation and oxidative stress by modulating the Nrf2-HO-1-NF-κB signaling pathway [27]. Similarly, GA has been shown to exert anti-inflammatory, antioxidative stress, and nephroprotective effects against paraquat-induced renal injury in male rats [18]. A traditional Chinese medicine preparation, the Tibetan Medicine Formula Jikan Mingmu Eye Drops, contains GA and has been shown to ameliorate dry eye syndrome in diabetic db/db mice [28]. In addition, Alexander et al. demonstrated that antioxidants such as GA were effective at quenching ROS in human corneal epithelial cells (HCECs), indicating potential to protect the corneal epithelium from oxidative damage if GA is included in a lubricant eye drop [29]. However, the efficacy of GA and its mechanism of action in DED have yet to be investigated.

**Fig. 1:**
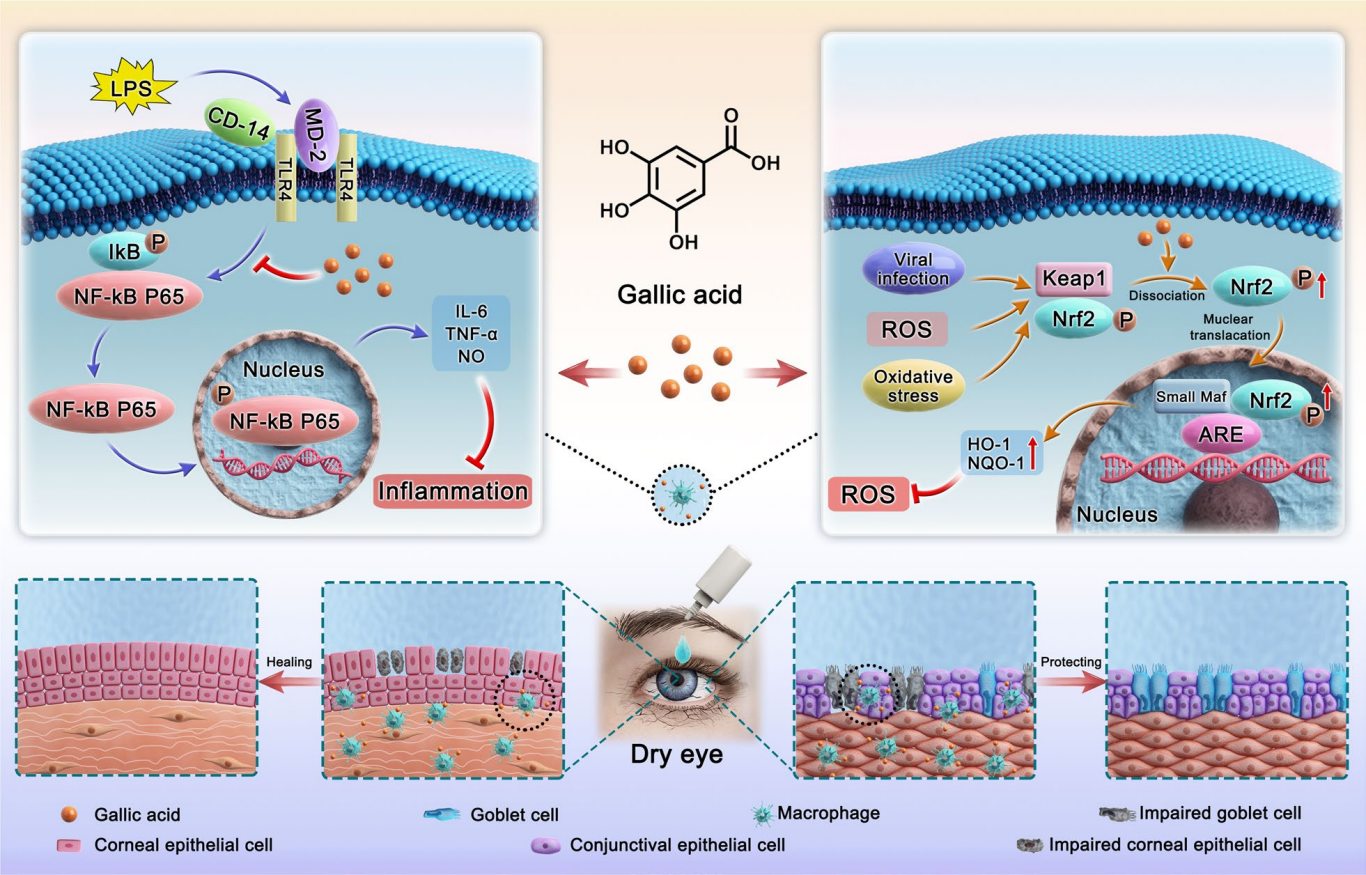
Schematic diagram of gallic acid treating dry eye through its anti-inflammatory and antioxidant effects. LPS, lipopolysaccharide; CD-14, clusterdifferentiation-14; MD-2, myeloid differentiation protein-2; TLR4, toll like receptor 4; IκB, inhibitor of NF-κB; P, phosphorylated; NF-κB P65, nuclear factor kappa-B P65; IL-6, interleukin 6; TNF-α, tumor necrosis factor alpha; NO, nitric oxide; ROS, reactive oxygen species; Keap1, Kelch-like ECH-associated protein 1; Nrf2, nuclear factor E2-relate factor-2; ARE, antioxidant response elements; HO-1, heme oxygenase-1; NQO-1, NADPH quinineoxidoreductase-1

Based on the pathogenesis of dry eye, combined with the strong anti-inflammatory and antioxidant capacity of GA, we hypothesized that GA has a potential effect on the treatment of dry eye. To verify this speculation, we investigated the underlying molecular mechanisms in lipopolysaccharide (LPS)-activated RAW264.7 macrophages and hypertonic-activated HCECs in vitro and evaluated the in vivo therapeutic efficacy in a mouse model of experimental dry eye (EDE). As shown in Fig. 1, an in vitro study demonstrated that GA inhibited the activation and nuclear translocation of inflammatory responses (e.g., NF-κB) and attenuated the production of various inflammatory mediators such as nitric oxide (NO) and pro-inflammatory cytokines (e.g., IL-6 and TNF-α). GA also promoted the activation and nuclear translocation of antioxidant responses such as nuclear factor E2-relate factor-2 (Nrf2), thereby inhibiting ROS production. In addition, an in vivo study showed that GA exhibited superior therapeutic efficacy against EDE by inhibiting the influx of inflammatory cytokines and protecting conjunctival goblet cells and corneal epithelial cells. Therefore, GA has potential for treating DED in clinics.

## Materials and methods

### Materials

Gallic acid (purity ≥ 99%) was purchased from J&K Chemicals CO (Beijing, China). LPS and epidermal growth factor were obtained from Sigma-Aldrich (St. Louis, MO, USA). HCECs and RAW264.7 macrophages were obtained from the American Type Culture Collection (Manassas, VA, USA). Insulin was obtained from Gibco (St. Louis, MO, USA). The 2',7'-Dichlorodihydrofluorescein diacetate (DCFH-DA) and cell counting kit-8 were acquired from Beyotime Biotechnology (Nanjing, China). The IL-6 (DY406) and TNF-α (DY410) DuoSet enzyme-linked immunosorbent assay (ELISA) kits were purchased from R&D Systems (Minneapolis, MN, USA). Phosphatase inhibitor was obtained from EpiZyme (Shanghai, China). Antibodies against p-P65 (#3033S, 1:1000), P65 (#8242S, 1:1000), phospho-IκB-α (#2859S, 1:1000), IκB-α (#4814S, 1:1000), β-actin (#3700, 1:5000), HO-1 (#48768, 1:1000), and NQO-1 (#48768, 1:1000) were purchased from Cell Signaling Technology (Danvers, MA, USA). Nrf2 (ab137550, 1:1000) and phospho-Nrf2 (ab76026, 1:1000) antibodies were obtained from Abcam (Cambridge, UK). Antibodies against NF-κB P65 (66535, 1:200), Nrf2 (16396, 1:200), and fluorescein (FITC) (SA00003, 1:100) were purchased from Proteintech (Chicago, IL, USA). Hematoxylin–eosin (H&E) and periodic acid-Schiff (PAS) staining kits were obtained from Solarbio Life Sciences (Beijing, China). An in situ cell death detection kit was obtained from Roche (Mannhein, Germany). The BCA kit, Griess reaction assay kit, antifade mounting medium with DAPI and protease inhibitor cocktail were obtained from Beyotime Biotechnology (Shanghai, China).

### Cell culture

HCECs were cultured in Dulbecco’s modified Eagle’s Medium/F12 (DMEM/F12) supplemented with 10% fetal bovine serum (FBS), 1% penicillin/streptomycin, 10 ng/mL epidermal growth factor, and 100 ng/mL insulin. RAW264.7 macrophages were cultured in DMEM supplemented with 1% penicillin/streptomycin and 10% FBS.

### In vitro hyperosmotic stress model and inflammation model

To test drug efficacy in vitro, we established two cell models related to the pathogenesis of DED. First, we simulated the state of HCECs in a hypertonic environment by adding sodium chloride (NaCl) to the culture medium. After stable subculture, HCECs were seeded onto a 6-well plate and switched to serum-free medium (DMEM/F12 without FBS) for 24 h. We cultured the cells in the medium for another hour by increasing the osmolality to 500 mOsm by adding an additional 90 mM NaCl to a serum-free medium with an osmolality of 320 mOsm. In addition, cells cultured in 500 mOsm medium were pre-treated with or without 100 μM GA (dissolved directly in the serum-free medium), which was added 23 h prior to NaCl supplementation.

We then stimulated macrophages with LPS to establish a classical model of inflammation. The RAW264.7 cells were seeded onto a 6- or 24-well plate and cultured in DMEM containing 10% FBS to achieve cell monolayers that were approximately 60–70% confluent after stable subculture. Subsequently, the cells were treated with 100 μM GA (dissolved directly in DMEM containing 10% FBS) for 1 h (ELISA and Griess reaction assay) or 16 h (fluorescence assay and Western blot analysis), followed by stimulation with 1 μg/mL LPS. Finally, P65 and p-P65 protein expression, phosphorylated-IkB-α and IkB-α activities were determined after 8 h of intracellular incubation by fluorescence assay and Western blot analysis. Release of IL-6, TNF-α and NO were measured in cell culture medium supernatant after 24 h incubation with ELISA and Griess reaction assay.

### In vitro cytotoxicity test

A cytotoxicity assay was performed to verify the safety of the drug in vitro. Briefly, after stable subculture, HCECs and RAW264.7 cells were seeded in a 96-well plate at a density of 5 × 10^4^ /mL and 1 × 10^5^ /mL in each well, respectively, and cell medium (100 μL per well) was added. The cells were then treated with a series of GA at concentrations ranging from 0 to 200 μM in a complete medium for 24 h. Thereafter, 10 μL of CCK-8 reagent was added to each well and cells were left to incubate at 37 ℃ for 1 h. Finally, the absorbance (A) was measured at 450 nm. Six parallel wells were used in each group, and the average value was obtained. At the same time, the control group included untreated cells containing complete media. No cells were set as the blank wells. The cell survival rate was calculated as follows: cell survival rate (%) = [(A_experimental group_ − A_blank group_) / (A_control group_ − A_blank group_)] × 100%. The experiment was repeated three times independently.

### Wound healing assay

We used a wound healing assay to demonstrate the effects of GA on cell migration in vitro. First, HCECs were cultured in DMEM/F12 containing 10% FBS. The cells were then plated in a 6-well plate and grown to 90% confluence. Thereafter, artificial wounds were created using a sterile 200 μL plastic pipette tip to scratch across the cell surface. Dissociated cells were removed by washing with phosphate buffered saline (PBS) (all PBS mentioned has a concentration of 0.01 M, pH = 7.4). The indicated amount of GA, at a final drug concentration of 0–100 μM was added for co-incubation, and images of the same area of the wound were taken at 0, 6, 18, and 24 h to determine wound closure. The scratch area was measured using Image J. The cell migration rate was expressed using the following formula: a = (1 − b/c) × 100%, where a is the migration rate, b is the area of scratch at the indicated time, and c is the initial area of the scratch.

### ELISA and detection of NO

In animal experiments, one sample consisted of the protein from the conjunctiva of two eyes and cornea of four eyes. In cell experiments, one sample consisted of the protein from the cell supernatant. The supernatant from each group was collected, and the TNF-α and IL-6 levels were quantified in accordance with the manufacturer’s protocols for the ELISA kits. The nitrite level was detected using the Griess reaction assay according to the manufacturer’s instructions.

### Measurement of cellular production of ROS

Intracellular ROS was detected by an oxidation-sensitive fluorescent probe (DCFH-DA). To detect the inhibitory effect of drugs on intracellular ROS production, RAW264.7 cells or HCECs were seeded onto 6-well plates at a density of 1 × 10^5^ /well and were incubated overnight. After drug addition, the cells were incubated for 16 h at 37 ℃, then LPS was added to each well and incubated for 8 h, hyperosmotic stress was added to each well and incubated for 1 h. Cells were then cultured in a serum-free DMEM/F12 or DMEM medium containing 10 μL DCFH-DA at 37 °C for 30 min and washed three times with PBS. DCFH-DA can be deacetylated intracellularly by nonspecific esterases and further oxidized by ROS to the fluorescent compound 2,7-dichlorofluorescein (DCF). Finally, DCF fluorescence was observed under a fluorescence microscope (Leica Microsystems, Mannheim, Germany), and the intensity of fluorescence was measured by flow cytometry (Becton Dickinson).

### Cellular immunofluorescence

Cells were attached to cell slides in 24-well plates. After 24 h of cell growth, GA was added to the medium to achieve a final concentration of 100 μM for 16 h. Next, LPS was added to each well, and incubated for 8 h. Then, the cell slides were fixed in 4% PFA for 20 min at room temperature and permeabilized with 0.1% Triton X-100 for 10 min at 4 ℃. Next, the cell slides were incubated with primary antibodies against NF-κB P65 (1:200 dilution) and Nrf2 (1:200 dilution) overnight at 4 ℃. After washing with PBS, cell slides were incubated with fluorescent secondary antibodies (1:400 dilution). Finally, after PBS washing, an antifade mounting medium containing DAPI (no washing required) was used and the slides sealed. Fluorescence images were obtained using a fluorescence microscope (Leica Microsystems).

### Western blot analysis

Briefly, the cells were lysed in Radio Immunoprecipitation Assay (RIPA) buffer containing a protease inhibitor cocktail and phosphatase inhibitor. Protein levels were quantified using a Bicinchoninic Acid Assay (BCA) kit, and then the loading buffer was added to the sample, which was boiled at 95 °C for 5 min. Equal amounts of protein were separated by sodium dodecyl sulfate polyacrylamide gel electrophoresis (SDS-PAGE) and transferred onto polyvinylidene fluoride (PVDF) membranes. Then, the blots were washed with TBST (10 mM Tris–HCl, 150 mM NaCl, 0.1% Tween-20, pH 7.6), blocked with 5% skimmed milk for 2 h, and incubated at 4 °C overnight with primary antibodies at the dilutions recommended by the supplier. β-actin acted as the loading control. Membranes were washed with TBST and incubated with secondary antibodies for 2 h at room temperature. Protein bands were detected using enhanced chemiluminescence (ECL) chemiluminescence reagents (Millipore) and visualized using a luminescent image analyzer. Finally, band analyses were performed using Image J [30].

### Experimental dry eye murine model and the experimental group

The animal research protocol was approved by the Laboratory Animal Ethics Committee of Wenzhou Medical University (ID No. wydw2022-0064). All procedures were performed in accordance with the Association of Research and Vision in Ophthalmology (ARVO) statement. One hundred and twenty female C57BL/6 mice, aged 6–8 weeks, were used in the following experiments. We created the EDE animal model by subcutaneous injection of 0.5 mg/0.2 mL scopolamine three times a day (8 a.m., 1 p.m., 6 p.m.) and exposed the animals to an intelligently controlled environmental system (ICES) with ventilation and 20% humidity as previously described [31, 32]. Food, water, and animal behavior were not restricted during the experiment.

To explore the concentration of GA in animal experiments, we reviewed the literature and found that the concentration of GA in the Tibetan Medicine Formula Jikan Mingmu Drops for db/db mice was 13.8830 mg/mL [28]. Therefore, we explored the protective effect of 1 mg/mL, 5 mg/mL and 10 mg/mL GA on corneal epithelial cells in EDE, as shown in Additional file 1: Figs. S5a and b, 5 mg/mL and 10 mg/mL GA can significantly reduce the spotting of corneal fluorescein sodium in mice, while 1 mg/mL GA has no effect. Hence, the lowest concentration of 5 mg/mL was chosen with good effect in subsequent experiments.

In order to explore the therapeutic effects of GA, after the preliminary screening to exclude existing ocular surface diseases, we randomly assigned a control group, named (1) Treatment group of the normal control group (T-NC), mice that did not receive EDE and were not given eye drops. The remaining mice were subjected to EDE for 10 days. After 10 days of dry eye modelling, the mice were randomized into three groups and continued with induction of EDE or treatment with eye drops for 5 days. The groupings were as follows: (2) Treatment group of the EDE group (T-EDE), mice that continued to receive EDE but were not given eye drops; (3) Treatment group of the EDE + PBS group (T-EDE + PBS), mice that continued to receive EDE and were treated with PBS eye drops; (4) Treatment group of the EDE + GA group (T-EDE + GA), mice that continued to receive EDE and were treated with eye drops made of 5 mg/mL GA dissolved in PBS. Eye drops (2 μL) were applied topically to both eyes of the mice three times a day (8 a.m., 1 p.m., and 6 p.m.) until they were euthanized by pentobarbital injection after 5 days.

To explore the preventive effect of GA on dry eye, after the preliminary screening to exclude existing ocular surface diseases, the mice were randomized into four groups for EDE-induced or eye drop treatment for five days. The groupings were as follows: (1) Prevention group of the normal control group (P-NC), mice that were not EDE-induced and not given eye drops; (2) Prevention group of the EDE group (P-EDE), mice that were EDE-induced but were not given eye drops; (3) Prevention group of the EDE + PBS group (P-EDE + PBS), mice that were EDE-induced and treated with PBS eye drops; (4) Prevention group of the EDE + GA group (P-EDE + GA), mice that were EDE-induced and were treated with 5 mg/mL GA dissolved in PBS eye drops. Eye drops (2 μL) were applied topically to both eyes of the mice three times a day (8 a.m., 1 p.m., and 6 p.m.) until they were euthanized by pentobarbital injection after five days.

### Corneal fluorescein staining

Corneal fluorescein staining score measurements were performed as previously described [33]. Sodium fluorescein (1%, 1 μL) was instilled into the inferior conjunctival sac using a micropipette. After 90 s, punctate staining of the corneal surface was performed in a double-blind fashion. The intensity of corneal fluorescein staining was calculated using a 4-point system: 0 point, no staining; 1 point, superficial stippling micropunctate staining with less than 30 spots; 2 points, punctate staining with 30 or more spots, but no diffuse staining; 3 points, severe diffuse staining, but no positive plaque or patch; and 4 points, positive fluorescein plaque or patch. The scores in the top, bottom, left, and right areas were totaled to generate a final score, which ranged from 0 to 16.

### Terminal deoxynucleotidyl transferase mediated dUTP nick end labeling (TUNEL) assay

After euthanasia, the eyeballs of the mice were removed and placed in optimum cutting temperature (OCT) glue at − 80℃ overnight. Frozen sections (sagittal plane, thickness of 10 μm) were obtained and placed at room temperature for approximately 1 h. Cell apoptosis in these tissue sections was examined by TUNEL staining using the in situ cell death detection kit according to the manufacturer’s instructions. Corneal sections were fixed with 4% paraformaldehyde at room temperature for 10 min. After fixation, they were permeabilized with Triton-X (0.1% in PBS) for 10 min, then 50 μL (5 μL enzyme solution in 45 μL solution) TUNEL reaction mixture was applied and incubated for 1 h at 37 °C in a humidified chamber. Counterstaining with DAPI (1:1000 dilution) was performed for 30 min. The sections were mounted with an anti-fading mounting medium and sealed with cover glass for microscopic observation.

### H&E staining and PAS staining

After 5 days of treatment, the entire lacrimal eyeball, with eyelids attached, and conjunctiva were fixed in 10% formalin for 24 h. After dehydration, the specimens were embedded in paraffin, cross-sectioned, and stained with H&E reagent and PAS for histological examination. Each section was observed under a microscope (Imager.z1; Germany). To prevent experimental bias, all images were captured randomly and assessed by two independent researchers in a blinded manner. Goblet cells in the superior conjunctiva were counted using three images taken from three mice at a magnification of 10 × the actual size.

### In vivo chronic ocular irritancy test and intraocular pressure monitoring

The chronic ocular irritancy of GA was assessed using ocular irritancy tests adapted and modified from previous studies [30]. Three female New Zealand albino rabbits (weight: ~ 2.5 kg) were acquired from Wenzhou Medical University Animal Center and housed individually in cages on a standard laboratory diet. Briefly, 50 μL of GA (5 mg/mL) was instilled into the lower conjunctival sac of the right eye of each rabbit, while 50 μL of PBS was instilled in the opposite eye as a reference; these two eye drops were applied three times a day, one drop each time. The eye drops were applied for seven days. The eyes were evaluated for clinical signs, intraocular pressure, and sodium fluorescein staining by an experienced doctor using a slit-lamp (Kang Hua®, Chongqing, China) before instilling eye drops every day. After 7 days and 24 h, or 48 h, or 72 h later, fluorescein sodium staining, intraocular pressure measurement, and evaluation of clinical signs were also done, and the number of infiltrates were scored from 0 (no sign) to 3 (severe) according to the Draize test under the supervision of trained optometrists who have experience working with animals. The score was based on the following criteria: 0 points, no redness, inflammation, or excessive tearing; 1 point, slight redness, slight inflammation, and slight tearing; 2 points, moderate redness, moderate inflammation, and excessive tearing; 3 points, severe redness, inflammation, and excessive tearing. After 10 days, histopathological changes in the cornea were observed by H&E staining at 24 h. The schematic of the treatment plan and observation plan can be seen in Figure S3a in the supporting information.

### Statistical analysis

All biological experiments were repeated three times independently. Data are expressed as the mean ± standard deviation. Statistical analyses were performed with SPSS 25 (SPSS Inc., Chicago, IL) using one-way analysis of variance (ANOVA) followed by post-hoc Tukey's test. A *P *value of 0.05 or less was considered statistically significant. *P < 0.05, **P < 0.01, ***P < 0.005, ****P < 0.001 compared to the LPS group or EDE group.

## Results

### Cytotoxicity of GA in HCECs and RAW264.7 cells

To investigate the cytotoxicity of GA, HCECs and RAW264.7 cells were cultured with various concentrations of GA for 24 h. As illustrated in Additional file 1: Fig. S1a and b, GA at a concentration below 100 μM had no significant cytotoxicity against both HCECs and RAW264.7 cells. This result indicated that the concentration used in this experiment was not cytotoxic.

### Effect of GA on wound healing assay

The effects of drugs on cell migration are closely associated with corneal healing. We measured the scratch area at the indicated times using Image J, and then evaluated the effect of GA on the HCEC migration rate in vitro. As illustrated in Additional file 1: Fig. S2, HCECs migration in the GA (10–100 μM GA) group was similar to that in the control group (0 μM GA), and there was no statistical difference between the GA group and the control group at 6 h, 18 h, and 24 h, suggesting that treatment with GA at concentrations up to 100 μM did not delay HCECs migration.

### In vivo chronic ocular irritancy test and the effect on intraocular pressure

During the development of ophthalmic drugs, the Draize test is often used to evaluate ocular irritation. GA eye drops (5 mg/mL) were topically instilled into rabbits three times a day for seven days. After one day of treatment, both the PBS and GA groups had mild corneal edema, conjunctival congestion, and slight tearing, but there was no significant difference between the two groups, and the symptoms subsided and returned to baseline after three days, as shown in Additional file 1: Fig. S3b and c. The explanation for this phenomenon is that rabbits, in the first three days of treatments such as irritation from eye drops, have a process of adaptation. As presented in Additional file 1: Fig. S3b and c, after 10 days, there was no eye irritation in the corneas of either group. After 10 days, H&E staining showed that the architecture of the cornea was clear and complete, and there was no corneal edema, neovascularization, or inflammatory cell infiltration. As shown in Additional file 1: Fig. S3d, continuous instillation of eye drops for seven days had no significant effect on the intraocular pressure of rabbits. The above experiments indicated that GA can be used on the ocular surface for seven days without obvious irritation symptoms, and GA had good ocular tolerance.

### In vitro antioxidant efficacy

**Fig. 2:**
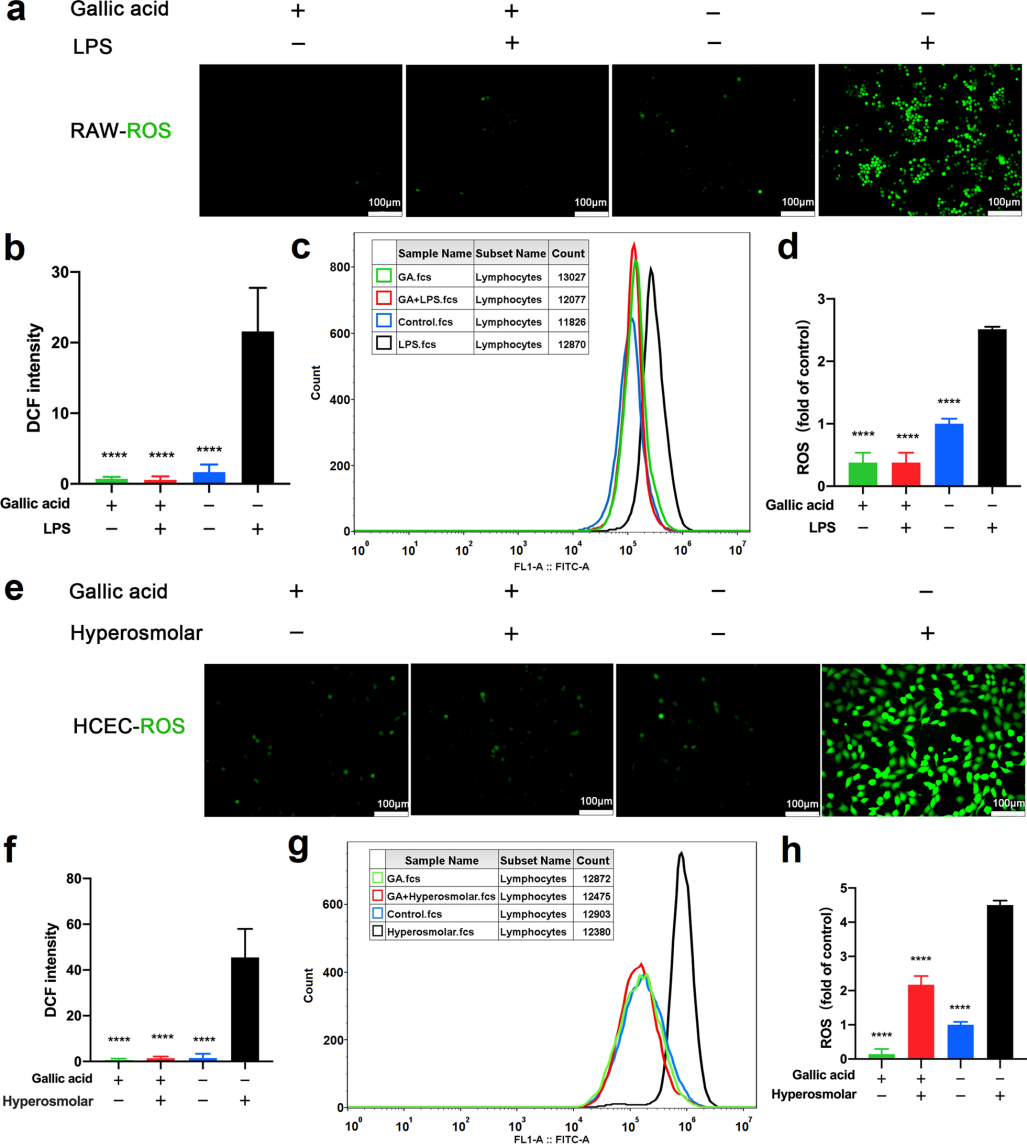
Gallic acid (GA) inhibits intracellular reactive oxygen species (ROS) production. **a** Green fluorescence indicates intracellular ROS in RAW264.7 with or without lipopolysaccharide (LPS). **b** Fluorescence statistics of RAW264.7 macrophages. **c** Flow cytometry assay quantified intracellular ROS in RAW264.7 cells. **d** Statistical results of flow cytometry. **e** Green fluorescence indicates intracellular ROS in human corneal epithelial cells (HCECs) with or without hypertonic stimulation. **f** Fluorescence data from (**e**). **g** Flow cytometry assay quantified intracellular ROS in HCECs. **h** Bar graphs of (**g**). RAW264.7 macrophages were pre-treated with GA (100 µM) for 16 h followed by the stimulation of LPS (1 µg/mL) for another 8 h. HCECs were pre-treated with GA (100 µM) for 23 h followed by the stimulation of sodium chloride solution (90 mM) for another 1 h. Data are presented as mean ± SD (n = 3). DCF, 2,7-dichlorofluorescein; FITC-A, fluorescein isothiocyanate-area; ****P < 0.001 compared to the LPS group

ROS play an important role in dry eye, and reducing ROS is an important treatment for DED [34]. To explore the immune cell response to inflammation in DED, we used a macrophage inflammation model. As shown in Fig. 2a and b, LPS significantly increased ROS in RAW264.7 cells, while GA significantly reduced ROS production. From Fig. 2c, d and Additional file 1: Fig. S4a, similar results were obtained by flow cytometry in RAW264.7 cells. In addition, hyperpermeability of tears is an important pathogenesis of dry eyes [4], and thus we used a hyperosmolarity model. In Fig. 2e and f, an osmolarity of 500 mOsm significantly increased the level of ROS in HCECs, and ROS in cells was significantly inhibited by GA. As shown in Fig. 2g, h and Additional file 1: Fig. S4b, the flow cytometry results also revealed that GA could inhibit ROS production in HCECs.

**Fig. 3:**
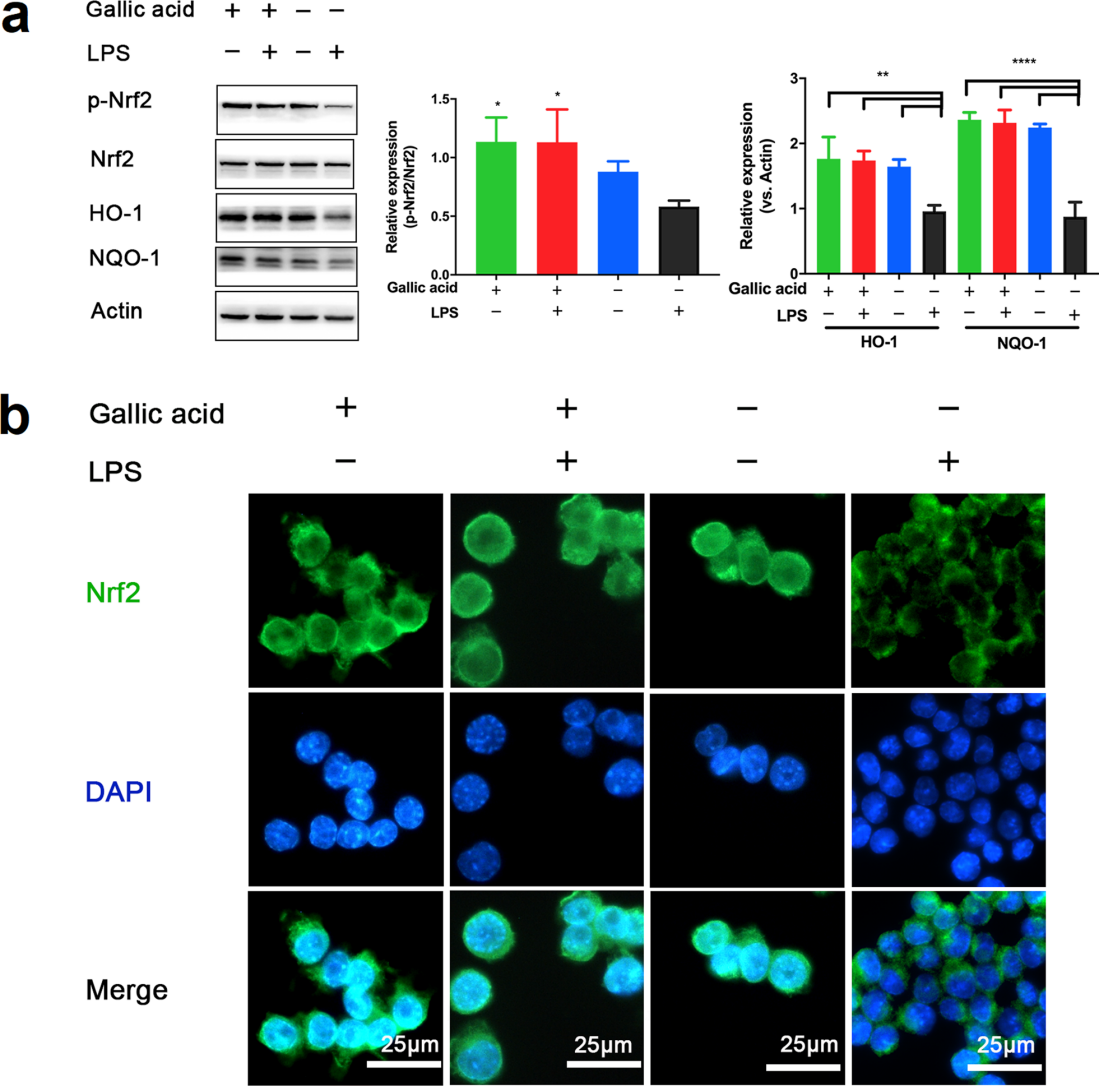
Antioxidant effect of gallic acid (GA) on lipopolysaccharide (LPS)-activated oxidative stress. **a** Western blot analysis of p-Nrf2, HO-1, and NADPH quinone oxidoreductase-1 (NQO-1) proteins in RAW264.7 macrophages. **b** Confocal microscopy images of RAW264.7 macrophages. Cell nuclei are shown in blue (DAPI) and nuclear factor E2-related factor 2 (Nrf2) is shown in green. RAW264.7 macrophages were pre-treated with GA (100 µM) for 16 h followed by the stimulation of LPS (1 µg/mL) for another 8 h. Data are presented as mean ± SD (n = 3). *P < 0.05, **P < 0.01, ****P < 0.001 compared to the LPS group

The transcription factor Nrf2 is highly sensitive to oxidative stress and protects cells by binding to the antioxidant response elements (AREs) in the nucleus [27]. The Nrf2 pathway was further quantified, as presented in Fig. 3a. Nrf2 and its downstream antioxidant enzymes heme oxygenase-1 (HO-1), and NADPH quinone oxidoreductase-1 (NQO-1) were upregulated after treatment with GA. Nrf2 activation was visually demonstrated by immunofluorescence, as shown in Fig. 3b, and nuclear translocation was more obvious after treatment with GA. These results suggest that GA promotes the activation of Nrf2, improves the expression of antioxidant enzymes, and can effectively inhibits ROS production.

### In vitro anti-inflammatory efficacy

**Fig. 4:**
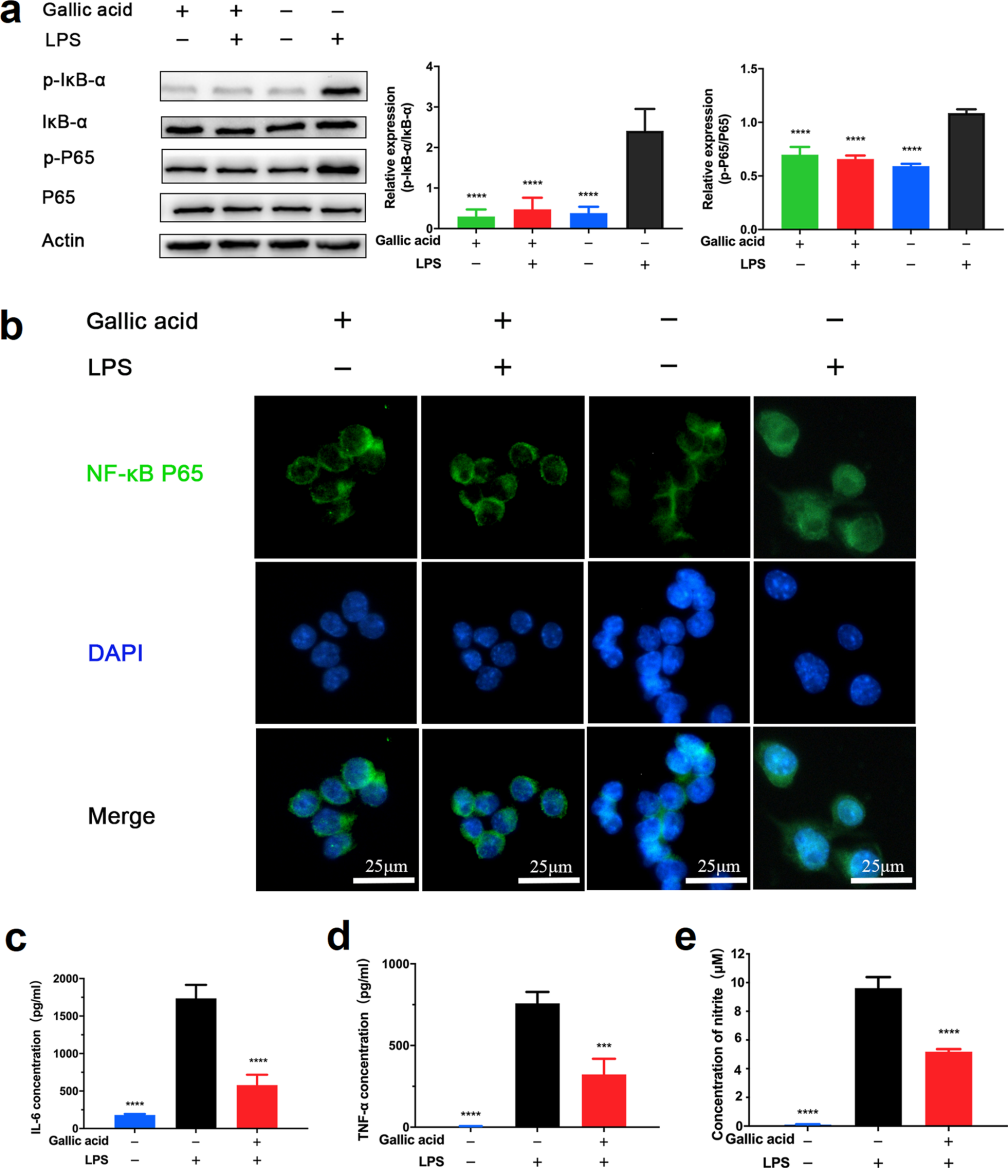
Gallic acid (GA) inhibits signaling pathways of nuclear transcription factor-κB (NF-κB) in lipopolysaccharide (LPS)-activated RAW264.7 macrophages. **a** Western blot analysis of p-P65 and p-IκB-α protein expression levels in RAW264.7 macrophages. **b** Confocal microscopy images of RAW264.7 macrophages. Cell nuclei are shown in blue (DAPI) and P65 is shown in green; RAW264.7 macrophages were pre-treated with GA (100 µM) for 16 h followed by the stimulation of LPS (1 µg/mL) for another 8 h. Data are presented as mean ± SD (n = 3). **c**–**e**, represent interleukin 6 (IL-6), tumor necrosis factor alpha (TNF-α), and nitric oxide (NO) in cell supernatant respectively. RAW264.7 macrophages were pre-treated with GA (100 µM) for 8 h followed by the stimulation of LPS (1 µg/mL) for another 16 h. ****P < 0.001 compared to the LPS group

A series of inflammatory reactions on the ocular surface contribute to the pathogenesis of dry eye, and how to effectively inhibit ocular surface inflammation has been a huge challenge. Therefore, we examined the anti-inflammatory effects of GA in a classic inflammatory model, LPS-activated RAW264.7 macrophages. As presented in Fig. 4a, Western blotting suggests that GA could effectively inhibit NF-κB pathway, by reducing the expression levels of phospho-IκB and p-P65 proteins. Further, the NF-κB pathway plays a transcriptional role in regulating phosphorylation (activation) and nuclear translocation of P65. As is shown in Fig. 4b, GA can significantly reduce the nucleation of P65. In addition, we further detected the expression of inflammatory factors IL-6, TNF-α, and NO by the Griess reaction assay [35] and ELISA kit. From Fig. 4c, d, and e, GA effectively inhibits the secretion of these inflammatory factors. Taken together, these results suggest that GA has the superior anti-inflammatory ability.

### Therapeutic effect of GA on EDE

**Fig. 5:**
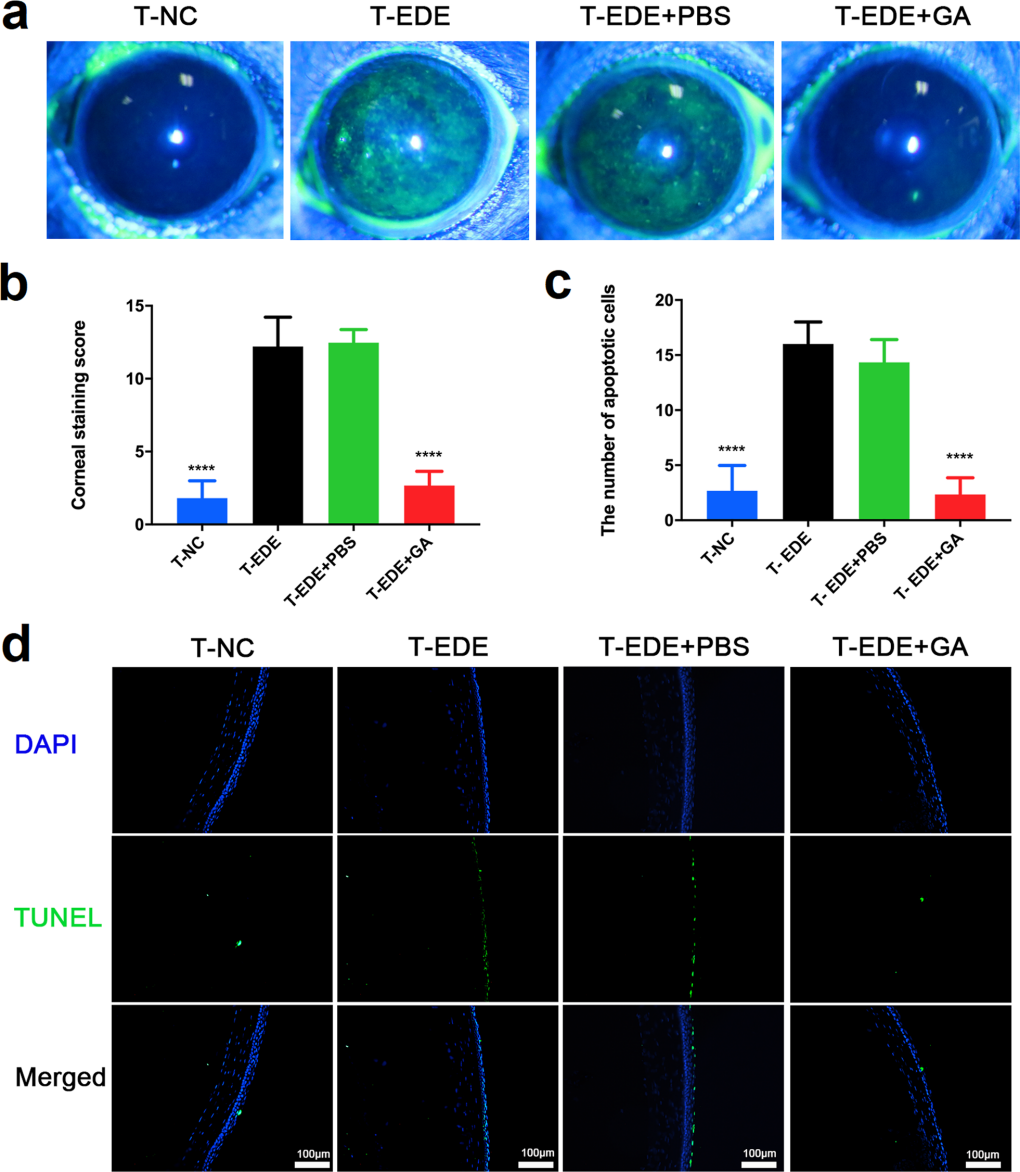
Gallic acid (GA) can effectively reduce corneal fluorescein sodium spot staining and cornea epithelial cell apoptosis. Corneal fluorescein representative figures (**a**) and staining scores (**b**) show the staining of the T-NC, T-EDE, T-EDE + PBS, and T-EDE + GA groups 15 days after desiccant stress. Apoptotic corneal epithelial cells count (**c**) and representative figures (**d**) show the apoptosis conditions of the T-NC, T-EDE, T-EDE + PBS, and T-EDE + GA groups 15 days after desiccant stress. The data are presented as mean ± SD (n = 5). ****P < 0.001 compared to the T-EDE group. EDE, experimental dry eye; PBS, phosphate buffered saline; T, therapeutic effect of drugs; T-NC, treatment group of the normal control group, not received EDE, not given eye drops; T-EDE, treatment group of the EDE group, received EDE, not given eye drops; T-EDE + PBS, treatment group of the EDE + PBS group, received EDE, given PBS eye drops; T-EDE + GA, treatment group of the EDE + GA group, received EDE, given GA eye drops

DED was induced in a mouse model by intraperitoneal injection of scopolamine in a dry and blowing environment. As shown in Fig. 5a, compared with the T-NC group, the fluorescein sodium staining on ocular surface in the T-DED group was significantly increased. There was no difference between the T-EDE + PBS and T-DED groups, while the sodium fluorescein staining of the T-EDE + GA group was significantly lower than that of the T-DED and T-EDE + PBS groups. In addition, apoptosis of corneal epithelial cells was examined by TUNEL staining (Fig. 5d), which showed similar results. These results suggest that GA protects the corneal epithelial cells in DED.

**Fig. 6:**
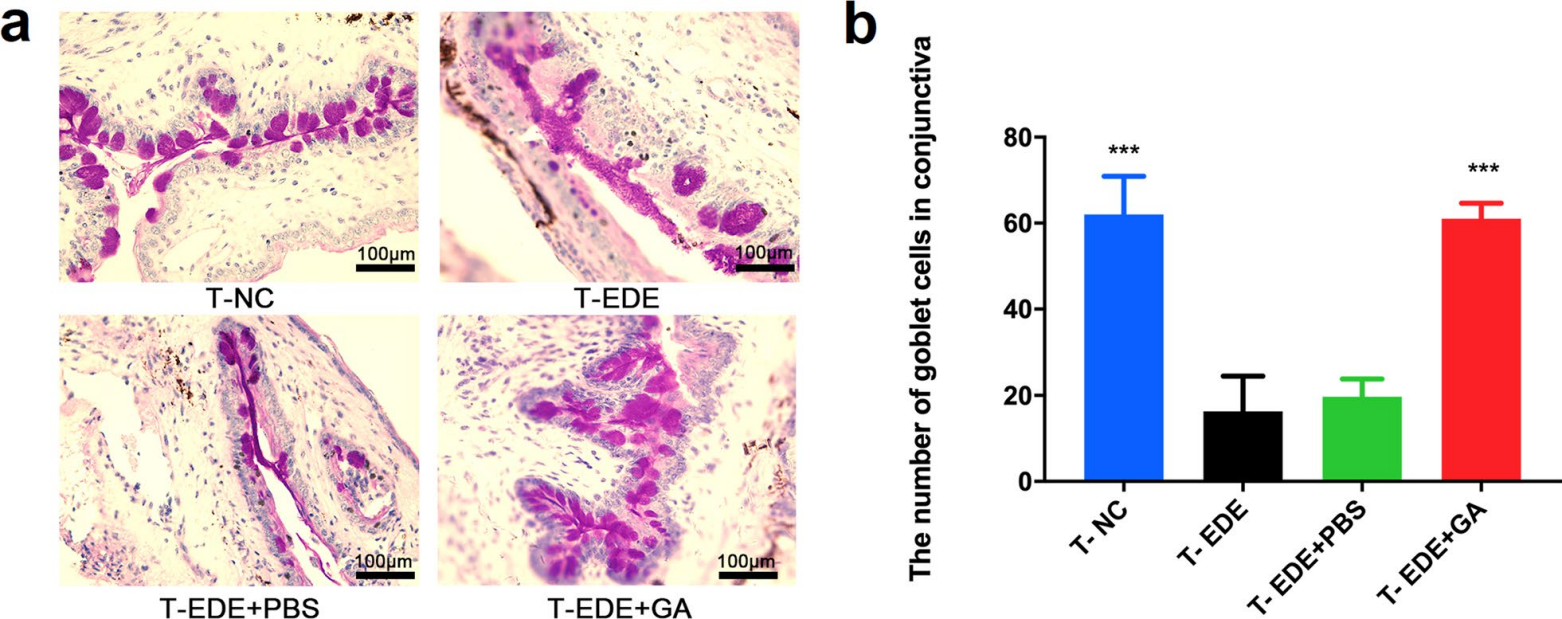
Gallic acid (GA) protects goblet cells. Goblet cells representative figures (**a**) and goblet cell count (**b**). The data are presented as mean ± SD (n = 3). ***P < 0.005 compared to the T-EDE group. Goblet cells were counted using three images taken from three mice at 10 × magnification. EDE, experimental dry eye; PBS, phosphate buffered saline; T-NC, treatment group of the normal control group, not received EDE, not given eye drops; T-EDE, treatment group of the EDE group, received EDE, not given eye drops; T-EDE + PBS, treatment group of the EDE + PBS group, received EDE, given PBS eye drops; T-EDE + GA, treatment group of the EDE + GA group, received EDE, given GA eye drops

To explore whether GA had a protective effect on conjunctival goblet cells, we removed the conjunctiva and performed PAS staining. When compared with the T-NC group, the conjunctival goblet cells of the T-DED group were significantly reduced (Fig. 6). Compared with the T-EDE group, the number of conjunctival goblet cells in the T-EDE + PBS group did not increase, while the conjunctival goblet cells in the T-EDE + GA group increased significantly compared with the T-EDE group and was similar to the T-NC group, indicating that GA can protect conjunctival goblet cells from reduction with DED.

**Fig. 7:**
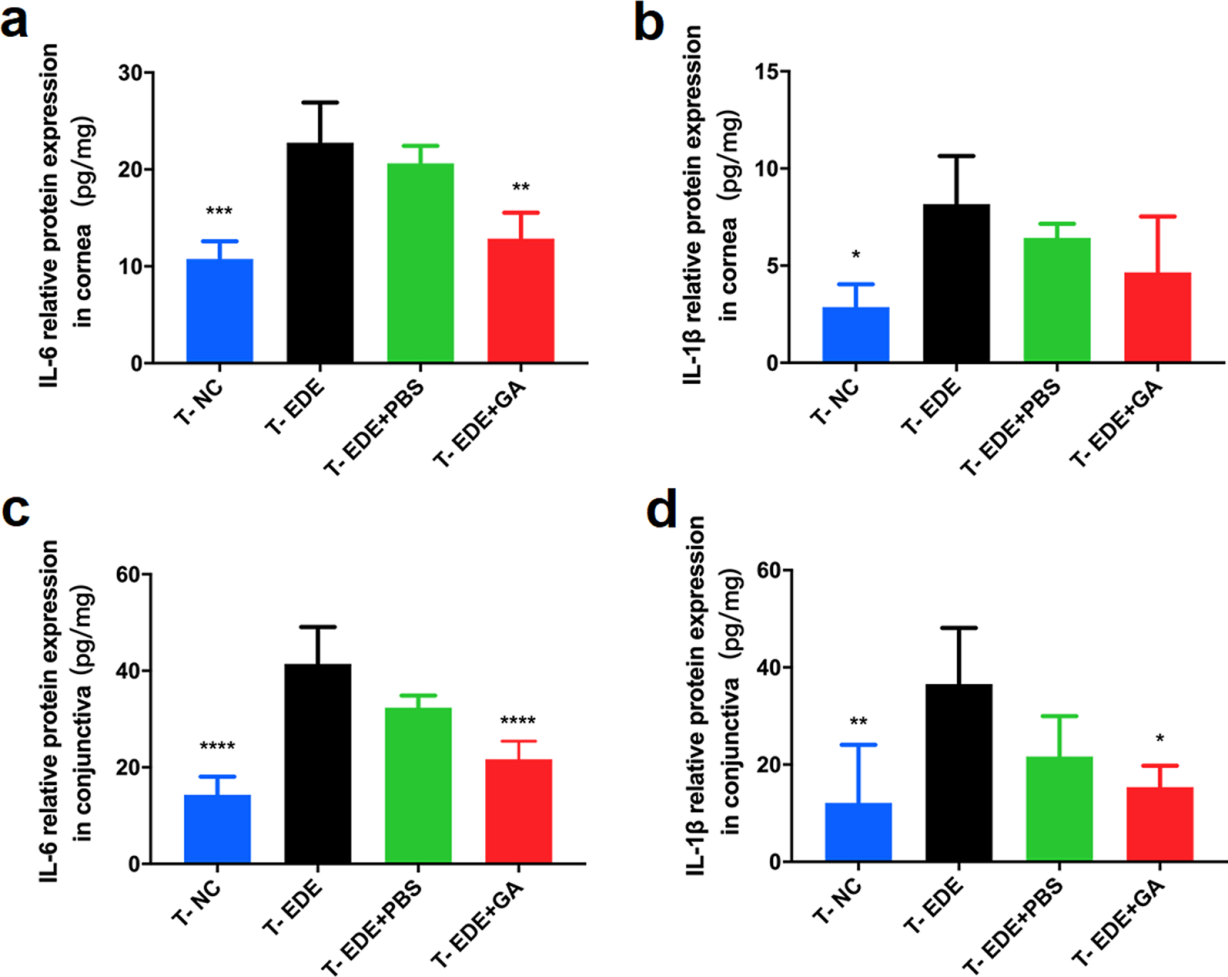
Gallic acid (GA) can effectively reduce the inflammatory factors in the cornea and conjunctiva. **a**, **b** respectively show IL-6 and IL-1β levels per mg of the cornea. **c**, **d** respectively show IL-6 and IL-1β levels per mg of the conjunctiva. The data are presented as mean ± SD (n = 4). *P < 0.05, **P < 0.01, ***P < 0.005 and ****P < 0.001 compared to the T-EDE group. EDE, experimental dry eye; PBS, phosphate buffered saline; T-NC, treatment group of the normal control group, not received EDE, not given eye drops; T-EDE, treatment group of the EDE group, received EDE, not given eye drops; T-EDE + PBS, treatment group of the EDE + PBS group, received EDE, given PBS eye drops; T-EDE + GA, treatment group of the EDE + GA group, received EDE, given GA eye drops

To further understand the therapeutic effects of GA, we measured the levels of inflammatory factors in the cornea and the conjunctiva. As shown in Fig. 7 in the T-DED group, the levels of inflammatory factors, including IL-6 and IL-1β, in the cornea and conjunctiva were greatly increased. Compared with the T-PBS group and T-EDE group, GA significantly reduced the levels of IL-6 in the cornea, and IL-6 and IL-1β in the conjunctiva. Although there was no statistically significant difference in IL-1β expression between the T-GA group and the T-EDE group in the cornea, a trending decrease was still observed. This may be because the cornea has relatively low levels of inflammatory factors, resulting in fewer significant differences between groups as compared to the conjunctiva. Taken together, GA can reduce the levels of inflammatory factors in the cornea and conjunctiva, thereby improving the symptoms and signs of DED.

DED is one of the most common reasons for seeking medical eye care, which can directly or indirectly increase health costs and reduce people's work productivity. DED can cause a significant financial burden [36], therefore the prevention of DED is particularly important. We also assessed the preventive effects of GA on DED. As shown in Additional file 1: Figs. S6, S7 and S8, prophylactic administration of GA significantly reduced staining and apoptosis of corneal epithelial cells, protected goblet cells and reduced the production of inflammatory factors in the corneal and conjunctiva. These suggest that GA have a good preventive effect on DED.

## Discussion

DED is a multifactorial disease of the ocular surface characterized by tear film instability and hyperosmolarity, ocular surface inflammation and damage, in which neurosensory abnormalities play etiological roles [1]. The pathogenic mechanisms underlying DED have not been fully elucidated, and the effects of inflammation and oxidative stress on the occurrence and development of DED, as well as their interaction, play an important role in the pathogenesis of this disease [37–39]. Recently, a variety of antioxidant drugs have shown therapeutic efficacy in relieving DED [40–45]. Nevertheless, the efficiency of GA in DED has not yet been comprehensively evaluated. This research suggests that GA could be used as a drug with few side effects with excellent anti-inflammatory and antioxidant properties, breaking the vicious cycle and preventing the development of DED.

In the use of eye drops for patients with dry eye, corneal epithelial cells first contact the eye drops, macrophages and other inflammatory cells migrate to the ocular surface [46]. Therefore, HCECs and RAW264.7 macrophages were selected to verify that GA in the 0–100 µM range was not toxic to these two cell types. Disruption of the corneal epithelial barrier occurs during the development of DED, and corneal epithelial cells proliferate and migrate during repair of the corneal epithelial barrier. Based on this process, we conducted a wound-healing assay, showing that GA at concentrations up to 100 μM does not affect the migration of corneal epithelial cells [1]. Moreover, dry eye is a chronic disease and patients often need to use eye drops for a long time; therefore, we conducted a chronic eye stimulation experiment in rabbits [47]. Compared with the acute eye stimulation test, this test can better reflect the possible toxicity of eye drops during real use. During testing, GA eye drops exhibited good long-term biocompatibility. In addition, long-term use of GA does not affect intraocular pressure.

The core of the DED etiology is the vicious cycle where instability and hyperosmolarity of the tear film trigger an inflammatory cascade, leading to ocular surface damage and further loss of tear film homeostasis [48]. Specifically, the increased osmotic pressure of tears, caused by reduced tear secretion or increased tear evaporation, and hypertonic tear film stimulates the production of excess ROS in the cornea and conjunctiva, which activates downstream inflammatory pathways to initiate inflammatory reactions on the ocular surface [49–52]. This pathway reduces both the production of ROS and the inflammatory response in tissues, which are both essential in preventing the progression of DED. It has been proposed that the oxidation/antioxidant imbalance is a central pathological process in the pathophysiology of DED [38, 53, 54]. In dry eyes, ROS can be produced not only in HCECs with hypertonic stimulation but also in macrophages that migrate to the ocular surface, and ROS can affect the function of macrophages. Therefore, we constructed a hypertonic-stimulated HCECs model and LPS-stimulated RAW264.7, a macrophage inflammatory model, and verified that GA has an excellent ability to inhibit the production of ROS in both models. Yoon et al. evaluated the therapeutic effect of plant extracts on dry eyes, but they only tested ROS in terms of antioxidation [55]. We tested not only ROS, but also antioxidant pathways and related enzymes. The Kelch-like ECH-associated protein 1 (Keap1) and Nrf2 system can be used to monitor oxidative stress. Nrf2 has been demonstrated to play an antioxidative stress effect by regulating the expression of antioxidant proteins (HO-1, Prxs, and NQO-1) [56]. Our results demonstrate that GA may upregulate the Nrf2 pathway and antioxidant proteins in this pathway such as HO-1 and NQO-1, and thus enhance the ability of cells to resist oxidative stress and reduce intracellular ROS. The decrease of ROS and the increase of p-Nrf2 are very closely related to antioxidative processes, and GA plays a crucial role in promoting antioxidation, but whether GA reduces ROS directly or indirectly through the upregulation of p-Nrf2 needs further research. After 8 h of LPS treatment, the level of p-Nrf2 decreased in RAW264.7 cells, while the Nrf2 level remained unchanged, which is consistent with the findings of Deng et al. [57], while in other reports, the level of Nrf2 in the cell was elevated after LPS treatment for 24 h [58, 59]. We speculate that following severe oxidative stress, p-Nrf2 may first decrease, leading to a compensatory upregulation of extranuclear Nrf2 which enters the nucleus to increase Nrf2 levels at 24 h. Additional time-point experiments in the future may be warranted.

Inflammation damages the outer surface epithelium, which may cause changes in tear film instability, cornea and conjunctiva wettability, and deterioration of subjective symptoms due to ocular surface nerve damage [60]. In the innate immunity of dry eye, macrophages migrate to the ocular surface, release a large number of inflammatory factors through the NF-κB pathway, and play an important role in the inflammatory cascade [61]. The classical model of macrophage inflammation is induced by LPS. LPS stimulates TLR4, leading to activation of the NF-κB, MAPK, and IRF5 pathways through the connector molecule MyD88 [62]. GA has been shown to inhibit the activities of NF-κB and MAPK, subsequently inhibiting the release of inflammatory factors (TNF-α and IL-6) and other inflammatory mediators such as COX-2 and NO [18]. In our experiment, we demonstrated that GA could inhibit the release of inflammatory factors, including IL-6, TNF-α and NO, by inhibiting the NF-KB pathway in a macrophage inflammation model induced by LPS. Therefore, GA exerts anti-inflammatory and antioxidative effects through the aforementioned pathways.

The EDE animal model used in this study combines low relative humidity, high air flow, and cholinergic blockade to impair lacrimal gland secretion, which is a standard DED model and has been used to study the pathogenesis of DED and related potential therapies [63–66]. There is evidence of increased ocular surface hazards in patients with xerophthalmia along with epithelial cell death (e.g., apoptosis), epithelial shedding and renewal [4]. In this experiment, corneal epithelial fluorescein sodium spot staining and corneal epithelial cell apoptosis in DED mice were significantly increased, while spot staining and apoptosis were significantly decreased after GA eye drop treatment. Ralph et al. emphasized that conjunctival goblet cell loss is typical of all forms of DED [67]. In this experiment, we demonstrated that GA can effectively protect conjunctival goblet cells. In addition, GA effectively reduced the concentrations of inflammatory factors in the cornea and conjunctiva. In conclusion, GA showed a good therapeutic effect in vivo in an EDE model. Our verification showed that GA has an effective preventive impact on EDE. Our experiments also confirmed the long-term biocompatibility of GA, indicating that it could be developed as a drug for the prevention of dry eyes in the future.

Our study has certain limitations. Eye drops are the treatment of choice for DED, however, drug delivery through the anterior segment is limited owing to the unique physiology and anatomy of the eye, providing low bioavailability. The tear film is composed of three phases: outer oil, intermediate water, and inner mucin. The oily and aqueous phases represent another barrier for hydrophilic and hydrophobic drugs, respectively [68]. Therefore, compared with hydrophilic GA, amphiphilic drugs have higher ocular surface bioavailability. Low bioavailability of a drug can lead to the need for multiple dosing, increased side effects, and inconvenience to patients; however, with in vivo chronic eye irritation trials, we demonstrated that three doses a day did not lead to side effects of eye irritation and increased intraocular pressure. Drug delivery through nanoparticle systems has the added advantages of cell targeting, improved cell uptake, and increased drug bioavailability [69]. In addition, the interaction of cyclic peptide ligand C or sialic acid-binding peptide with corneal collagen or corneal epithelium can further increase the retention time of drugs on the ocular surface and enhance bioavailability [70, 71]. Therefore, in the near future, further drug modification or a suitable drug delivery system for GA should be developed to increase the bioavailability of GA. In addition, the stratified cultures of corneal epithelial cells are closer to the complete corneal epithelium [72], and the application of stratified cultures to the hypertonic cell model of HCECs can better respond to the effect of hyperosmolar stress on the cornea in vivo, and the therapeutic effect of GA in this model may be further tested in the future.

## Conclusion

GA has good long-term biocompatibility both in vitro and in vivo. Furthermore, GA achieves excellent anti-inflammatory and antioxidant effects through the NF-κB and Nrf2 pathways in vitro, reduces inflammation in the cornea and conjunctiva, and as well as protects corneal epithelial cells and conjunctiva goblet cells in EDE. Taken together, we demonstrated GA’s potential as a topical anti-inflammatory and antioxidant eye drop to prevent and treat DED.

## Supplementary Information

**Additional file 1: Figure S1.** The effect of pre-treatment with gallic acid (GA) on the viability of human corneal epithelial cells (HCECs) and RAW264.7 cells. The effect of GA on the viability of HCECs (**a**) and RAW264.7 cells (**b**). Data are presented as mean ± SD (n = 6). **P < 0.01, *P < 0.05 compared to the control group (0 μM gallic acid). **Figure S2.** Wound healing results. Control (**a**) and 100 μM gallic acid (**b**) on wound closure was visualized at 0 h post scratch of human corneal epithelial cell (HCEC) monolayer; Control (**c**) and 100 μM gallic acid (**d**) were 24 h after wound healing. **e** The percentage reduction of the average wound width at 6 h, 18 h and 24 h post scratch of HCEC monolayer. Data are presented as mean ± SD (n = 6). **Figure S3.** Gallic acid (GA) has good long-term biocompatibility. **a** Treatment plan and observation plan; **b** Ocular surface and conjunctiva images, fluorescein sodium staining of the cornea and hematoxylin and eosin (H&E) staining of the cornea after the eyes were treated with phosphate buffered saline (PBS) solution and gallic acid solution (5 mg/mL) at 10 days post instillation; **c** Draize Test score for ocular surface irritation over 10 days. **d** Intraocular pressure changes over 10 days. Data are presented as mean ± SD (n = 3). **Figure S4.** Representative images of the gate plot of intracellular reactive oxygen species (ROS) production in RAW264.7 (**a**) and human corneal epithelial cells (HCECs) (**b**). The production of ROS was measured by flow cytometry using the fluorescent probe 2’,7’-Dichlorodihydrofluorescein diacetate (DCFH-DA). SSC-A, side scatter-area; FITC-A, fluorescein isothiocyanate-area; GA, gallic acid; LPS, lipopolysaccharide. **Figure S5.** 5 mg/mL and 10 mg/mL gallic acid (GA) can significantly reduce corneal fluorescein sodium spotting. Corneal fluorescein representative figures (**a**) and staining scores (**b**) show the staining of the NC, EDE, EDE + 10 mg/mL GA, EDE + 5 mg/mL GA, EDE + 1 mg/mL GA groups on the fifth day after desiccant stress. The data are presented as mean ± SD (n = 3). NC, normal control, not received EDE, not given eye drops; EDE, experimental dry eye, received EDE, not given eye drops; EDE + 10 mg/mL GA, received EDE, given 10 mg/mL GA eye drops; EDE + 5 mg/mL GA, received EDE, given 5 mg/mL GA eye drops; EDE + 1 mg/mL GA, received EDE, given 1 mg/mL GA eye drops. ****P < 0.001 compared to the EDE group. **Figure S6.** Gallic acid (GA) can prevent corneal fluorescein sodium spot staining and cornea epithelial cell apoptosis. Corneal fluorescein representative figures (**a**) and staining scores (**b**) show the staining on the fifth day after desiccant stress. Apoptotic corneal epithelial cell count (**c**) and representative figures (**d**) show the apoptosis conditions 5 days after desiccant stress. The data are presented as mean ± SD (n = 6). NC, normal control; EDE, experimental dry eye; PBS, phosphate buffered saline; P, preventive effect of the drug; P-NC, not received EDE, not given eye drops; P-EDE: received EDE, not given eye drops; P-EDE + PBS: received EDE, given PBS eye drops; P-EDE + GA: received EDE, given GA eye drops. ****P < 0.001 compared to the P-EDE group. **Figure S7.** Gallic acid (GA) protects goblet cells. Goblet cells representative figures (**a**) and goblet cell count (**b**). The data are presented as mean ± SD (n = 3). NC, normal control; EDE, experimental dry eye; PBS, phosphate buffered saline; P: preventive effect of the drug; P-NC: not received EDE, not given eye drops; P-EDE: received EDE, not given eye drops; P-EDE + PBS: received EDE, given PBS eye drops; P-EDE + GA: received EDE, given GA eye drops. ***P < 0.005 compared to the P-EDE group. **Figure S8.** Gallic acid (GA) inhibits the elevation of inflammatory factors in the cornea and conjunctiva. **a, b** respectively show IL-6 and IL-1β levels per mg of the cornea. **c, d** respectively show IL-6 and IL-1β levels per mg of the conjunctiva. The data are presented as mean ± SD (n = 3). NC, normal control; EDE, experimental dry eye; PBS, phosphate buffered saline; P: preventive effect of the drug; P-NC: not received EDE, not given eye drops; P-EDE: received EDE, not given eye drops; P-EDE + PBS: received EDE, given PBS eye drops; P-EDE + GA: received EDE, given GA eye drops. **P < 0.01, *P < 0.05 compared to the P-EDE group.

## Data Availability

The datasets used and analyzed during the current study are available from the corresponding author on reasonable request.

## Abbreviations

DED: Dry eye disease
MAPK: Mitogen-activated protein kinase
NF-κB: Nuclear transcription factor-κB
IL-1: Interleukin 1
IL-6: Interleukin 6
TNF-α: Tumor necrosis factor alpha
MMP-9: Matrix metallopeptidase 9
ROS: Reactive oxygen species
NLRP3: Nucleotide-binding oligomerization domain, leucine-rich repeat and pyrin domain-containing 3
APCs: Antigen-presenting cells
GA: Gallic acid
HCECs: Human corneal epithelial cells
LPS: Lipopolysaccharide
EDE: Experimental dry eye
NO: Nitric oxide
Nrf2: Nuclear factor E2-relate factor-2
DMEM/F12: Dulbecco’s modified Eagle’s Medium /F12
FBS: Fetal bovine serum
NaCl: Sodium chloride
p-P65: Phosphorylated P65
ELISA: Enzyme-linked immunosorbent assay
DAPI: 4,6-Diamidino-2-phenylindole
SDS-PAGE: Sodium dodecyl sulfate polyacrylamide gel electrophoresis
PVDF: Polyvinylidene fluoride
ARVO: Association of Research and Vision in Ophthalmology
ICES: Intelligently controlled environmental system
TUNEL: Terminal deoxynucleotidyl transferase mediated dUTP nick end labeling
H&E: Hematoxylin and eosin
PAS: Periodic acid-Schiff
AREs: Antioxidant response elements
HO-1: Heme oxygenase-1
NQO-1: NADPH quinineoxidoreductase-1
Keap1: Kelch-like ECH-associated protein 1

## References

[1] Craig JP, Nichols KK, Akpek EK, Caffery B, Dua HS, Joo CK (2017). TFOS DEWS II definition and classification report. Ocul Surf.

[2] Stapleton F, Alves M, Bunya VY, Jalbert I, Lekhanont K, Malet F (2017). TFOS DEWS II epidemiology report. Ocul Surf.

[3] Galor A, Zheng DD, Arheart KL, Lam BL, Perez VL, McCollister KE (2012). Dry eye medication use and expenditures: data from the medical expenditure panel survey 2001 to 2006. Cornea.

[4] Bron AJ, de Paiva CS, Chauhan SK, Bonini S, Gabison EE, Jain S (2017). TFOS DEWS II pathophysiology report. Ocul Surf.

[5] Zheng Q, Ren Y, Reinach PS, Xiao B, Lu H, Zhu Y (2015). Reactive oxygen species activated NLRP3 inflammasomes initiate inflammation in hyperosmolarity stressed human corneal epithelial cells and environment-induced dry eye patients. Exp Eye Res.

[6] Wang HH, Chen WY, Huang YH, Hsu SM, Tsao YP, Hsu YH (2022). Interleukin-20 is involved in dry eye disease and is a potential therapeutic target. J Biomed Sci.

[7] You IC, Coursey TG, Bian F, Barbosa FL, de Paiva CS, Pflugfelder SC (2015). Macrophage phenotype in the ocular surface of experimental murine dry eye disease. Arch Immunol Ther Exp (Warsz).

[8] Stevenson W, Chauhan SK, Dana R (2012). Dry eye disease: an immune-mediated ocular surface disorder. Arch Ophthalmol.

[9] Wang YM, Ji R, Chen WW, Huang SW, Zheng YJ, Yang ZT (2019). Paclitaxel alleviated sepsis-induced acute lung injury by activating MUC1 and suppressing TLR-4/NF-κB pathway. Drug Des Devel Ther.

[10] Caban-Toktas S, Sahin A, Lule S, Esendagli G, Vural I, KarlıOguz K (2020). Combination of Paclitaxel and R-flurbiprofen loaded PLGA nanoparticles suppresses glioblastoma growth on systemic administration. Int J Pharm.

[11] Stolzenburg N, Breinl J, Bienek S, Jaguszewski M, Löchel M, Taupitz M (2016). Paclitaxel-coated balloons: investigation of drug transfer in healthy and atherosclerotic arteries—first experimental results in rabbits at low inflation pressure. Cardiovasc Drugs Ther.

[12] Yang FH, Zhang Q, Liang QY, Wang SQ, Zhao BX, Wang YT (2015). Bioavailability enhancement of paclitaxel via a novel oral drug delivery system: paclitaxel-loaded glycyrrhizic acid micelles. Molecules.

[13] Wu G, Cheng B, Qian H, Ma S, Chen Q (2019). Identification of HSP90 as a direct target of artemisinin for its anti-inflammatory activity via quantitative chemical proteomics. Org Biomol Chem.

[14] Wang X, Liu P, Wu Q, Zheng Z, Xie M, Chen G (2022). Sustainable antibacterial and anti-inflammatory silk suture with surface modification of combined-therapy drugs for surgical site infection. ACS Appl Mater Interfaces.

[15] Jiang Y, Pei J, Zheng Y, Miao YJ, Duan BZ, Huang LF (2022). Gallic acid: a potential anti-cancer agent. Chin J Integr Med.

[16] Guo P, Anderson JD, Bozell JJ, Zivanovic S (2016). The effect of solvent composition on grafting gallic acid onto chitosan via carbodiimide. Carbohydr Polym.

[17] Govea-Salas M, Rivas-Estilla AM, Rodríguez-Herrera R, Lozano-Sepúlveda SA, Aguilar-Gonzalez CN, Zugasti-Cruz A (2016). Gallic acid decreases hepatitis C virus expression through its antioxidant capacity. Exp Ther Med.

[18] Nouri A, Heibati F, Heidarian E (2021). Gallic acid exerts anti-inflammatory, anti-oxidative stress, and nephroprotective effects against paraquat-induced renal injury in male rats. Naunyn Schmiedebergs Arch Pharmacol..

[19] Liu KC, Huang AC, Wu PP, Lin HY, Chueh FS, Yang JS (2011). Gallic acid suppresses the migration and invasion of PC-3 human prostate cancer cells via inhibition of matrix metalloproteinase-2 and -9 signaling pathways. Oncol Rep.

[20] López-Lázaro M, Calderón-Montaño JM, Burgos-Morón E, Austin CA (2011). Green tea constituents (-)-epigallocatechin-3-gallate (EGCG) and gallic acid induce topoisomerase I- and topoisomerase II-DNA complexes in cells mediated by pyrogallol-induced hydrogen peroxide. Mutagenesis.

[21] Rajalakshmi K, Devaraj H, Niranjali DS (2001). Assessment of the no-observed-adverse-effect level (NOAEL) of gallic acid in mice. Food Chem Toxicol.

[22] Werner RA, Rossmann A, Schwarz C, Bacher A, Schmidt HL, Eisenreich W (2004). Biosynthesis of gallic acid in Rhus typhina: discrimination between alternative pathways from natural oxygen isotope abundance. Phytochemistry.

[23] Bai J, Zhang Y, Tang C, Hou Y, Ai X, Chen X (2021). Gallic acid: pharmacological activities and molecular mechanisms involved in inflammation-related diseases. Biomed Pharmacother.

[24] Bensaad LA, Kim KH, Quah CC, Kim WR, Shahimi M (2017). Anti-inflammatory potential of ellagic acid, gallic acid and punicalagin A&B isolated from Punica granatum. BMC Complement Altern Med.

[25] Tanaka M, Sato A, Kishimoto Y, Mabashi-Asazuma H, Iida K (2020). Gallic Acid inhibits lipid accumulation via AMPK pathway and suppresses apoptosis and macrophage-mediated inflammation in hepatocytes. Nutrients.

[26] Variya BC, Bakrania AK, Madan P, Patel SS (2019). Acute and 28-days repeated dose sub-acute toxicity study of gallic acid in albino mice. Regul Toxicol Pharmacol.

[27] Sohrabi F, Dianat M, Badavi M, Radan M, Mard SA (2021). Gallic acid suppresses inflammation and oxidative stress through modulating Nrf2-HO-1-NF-κB signaling pathways in elastase-induced emphysema in rats. Environ Sci Pollut Res Int.

[28] Ai X, Hou Y, Wang X, Wang X, Liang Y, Zhu Z (2019). Amelioration of dry eye syndrome in db/db mice with diabetes mellitus by treatment with Tibetan Medicine Formula Jikan Mingmu Drops. J Ethnopharmacol.

[29] Stoddard AR, Koetje LR, Mitchell AK, Schotanus MP, Ubels JL (2013). Bioavailability of antioxidants applied to stratified human corneal epithelial cells. J Ocul Pharmacol Ther.

[30] Yan J, Zhang Y, Wang L, Zhao L, Tang S, Wang Y (2022). TREM2 activation alleviates neural damage via Akt/CREB/BDNF signalling after traumatic brain injury in mice. J Neuroinflamm.

[31] Yoon KC, Ahn KY, Choi W, Li Z, Choi JS, Lee SH (2011). Tear production and ocular surface changes in experimental dry eye after elimination of desiccating stress. Invest Ophthalmol Vis Sci.

[32] Chen W, Zhang X, Zhang J, Chen J, Wang S, Wang Q (2008). A murine model of dry eye induced by an intelligently controlled environmental system. Invest Ophthalmol Vis Sci.

[33] Sung MS, Li Z, Cui L, Choi JS, Choi W, Park MJ (2015). Effect of topical 5-aminoimidazole-4-carboxamide-1-β-d-ribofuranoside in a mouse model of experimental dry eye. Invest Ophthalmol Vis Sci.

[34] Seen S, Tong L (2018). Dry eye disease and oxidative stress. Acta Ophthalmol.

[35] Oliveira S, Monteiro-Alfredo T, Henriques R, Ribeiro CF, Seiça R, Cruz T (2022). Improvement of glycaemia and endothelial function by a new low-dose curcuminoid in an animal model of type 2 diabetes. Int J Mol Sci.

[36] Moss SE, Klein R, Klein BE (2000). Prevalence of and risk factors for dry eye syndrome. Arch Ophthalmol.

[37] Li Q, Wu X, Xin S, Wu X, Lan J (2021). Preparation and characterization of a naringenin solubilizing glycyrrhizin nanomicelle ophthalmic solution for experimental dry eye disease. Eur J Pharm Sci.

[38] Dogru M, Kojima T, Simsek C, Tsubota K (2018). Potential role of oxidative stress in ocular surface inflammation and dry eye disease. Invest Ophthalmol Vis Sci..

[39] Yamaguchi T (2018). Inflammatory response in dry eye. Invest Ophthalmol Vis Sci..

[40] Park B, Jo K, Lee TG, Hyun SW, Jin SK, Kim CS (2019). Polydatin inhibits NLRP3 inflammasome in dry eye disease by attenuating oxidative stress and inhibiting the NF-κB pathway. Nutrients.

[41] Na YJ, Choi KJ, Jung WH, Park SB, Kang S, Ahn JH (2020). A novel selective 11β-HSD1 inhibitor, (E)-4-(2-(6-(2,6-dichloro-4-(trifluoromethyl)phenyl)-4-methyl-1,1-dioxido-1,2,6-thiadiazinan-2-yl)acetamido)adamantan-1-carboxamide (KR-67607), prevents BAC-induced dry eye syndrome. Int J Mol Sci.

[42] Pagar KR, Khandbahale SV, Phadtare DG (2019). The therapeutic potential of resveratrol: a review of clinical trials. Asian J Pharm Res.

[43] Bielory L, Tabliago NRA (2020). Flavonoid and cannabinoid impact on the ocular surface. Curr Opin Allergy Clin Immunol.

[44] Chakrawarti L, Agrawal R, Dang S, Gupta S, Gabrani R (2016). Therapeutic effects of EGCG: a patent review. Expert Opin Ther Pat.

[45] Anand David AV, Arulmoli R, Parasuraman S (2016). Overviews of biological importance of quercetin: a bioactive flavonoid. Pharmacogn Rev.

[46] Perez VL, Pflugfelder SC, Zhang S, Shojaei A, Haque R (2016). Lifitegrast, a novel integrin antagonist for treatment of dry eye disease. Ocul Surf.

[47] Liu H, Bi XQ, Wu YQ, Pan MM, Ma XH, Mo LH (2021). Cationic self-assembled peptide-based molecular hydrogels for extended ocular drug delivery. Acta Biomater.

[48] Gupta PK, Asbell P, Sheppard J (2020). Current and future pharmacological therapies for the management of dry eye. Eye Contact Lens.

[49] Zheng Q, Ren Y, Reinach PS, She Y, Xiao B, Hua S (2014). Reactive oxygen species activated NLRP3 inflammasomes prime environment-induced murine dry eye. Exp Eye Res.

[50] Ray P, Huang BW, Tsuji Y (2012). Reactive oxygen species (ROS) homeostasis and redox regulation in cellular signaling. Cell Signal.

[51] Lee S, Zheng M, Kim B, Rouse BT (2002). Role of matrix metalloproteinase-9 in angiogenesis caused by ocular infection with herpes simplex virus. J Clin Invest.

[52] Favero G, Moretti E, Krajčíková K, Tomečková V, Rezzani R (2021). Evidence of polyphenols efficacy against dry eye disease. Antioxidants (Basel).

[53] Ivanov IV, Mappes T, Schaupp P, Lappe C, Wahl S (2018). Ultraviolet radiation oxidative stress affects eye health. J Biophoton.

[54] Shoham A, Hadziahmetovic M, Dunaief JL, Mydlarski MB, Schipper HM (2008). Oxidative stress in diseases of the human cornea. Free Radic Biol Med.

[55] Choi W, Lee JB, Cui L, Li Y, Li Z, Choi JS (2016). Therapeutic efficacy of topically applied antioxidant medicinal plant extracts in a mouse model of experimental dry eye. Oxid Med Cell Longev.

[56] Ooi BK, Goh BH, Yap WH (2017). Oxidative stress in cardiovascular diseases: involvement of NRF2 antioxidant redox signaling in macrophage foam cells formation. Int J Mol Sci.

[57] Deng J, Lin DQ, Ding XY, Wang Y, Hu YH, Shi H (2022). Multifunctional supramolecular filament hydrogel boosts anti-inflammatory efficacy in vitro and in vivo. Adv Funct Mater..

[58] Du Y, Zhu S, Wang R, Chen X, Cai K (2022). Isolation and identification of anti-inflammatory peptide from goose blood hydrolysate to ameliorate lps-mediated inflammation and oxidative stress in RAW264.7 macrophages. Molecules.

[59] Sun S, Zhang J, Li H, Du Y, Li S, Li A (2021). Anti-inflammatory activity of the water extract of Chloranthus serratus roots in LPS-stimulated RAW264.7 cells mediated by the Nrf2/HO-1, MAPK and NF-κB signaling pathways. J Ethnopharmacol..

[60] Kojima T, Dogru M, Kawashima M, Nakamura S, Tsubota K. Advances in the diagnosis and treatment of dry eye. Prog Retin Eye Res. 2020;100842. 10.1016/j.preteyeres.2020.100842 32004729

[61] Chen Y, Dana R (2021). Autoimmunity in dry eye disease—an updated review of evidence on effector and memory Th17 cells in disease pathogenicity. Autoimmun Rev.

[62] Dorrington MG, Fraser IDC (2019). NF-κB signaling in macrophages: dynamics, crosstalk, and signal integration. Front Immunol.

[63] Krauss AH, Corrales RM, Pelegrino FS, Tukler-Henriksson J, Pflugfelder SC, De Paiva CS (2015). Improvement of outcome measures of dry eye by a novel integrin antagonist in the murine desiccating stress model. Invest Ophthalmol Vis Sci.

[64] de Paiva CS, Schwartz CE, Gjörstrup P, Pflugfelder SC (2012). Resolvin E1 (RX-10001) reduces corneal epithelial barrier disruption and protects against goblet cell loss in a murine model of dry eye. Cornea.

[65] Goyal S, Chauhan SK, Dana R (2012). Blockade of prolymphangiogenic vascular endothelial growth factor c in dry eye disease. Arch Ophthalmol.

[66] Lee HS, Chauhan SK, Okanobo A, Nallasamy N, Dana R (2011). Therapeutic efficacy of topical epigallocatechin gallate in murine dry eye. Cornea.

[67] Ralph RA (1975). Conjunctival goblet cell density in normal subjects and in dry eye syndromes. Invest Ophthalmol.

[68] Jumelle C, Gholizadeh S, Annabi N, Dana R (2020). Advances and limitations of drug delivery systems formulated as eye drops. J Control Release.

[69] Nagai N, Ishii M, Seiriki R, Ogata F, Otake H, Nakazawa Y (2020). Novel sustained-release drug delivery system for dry eye therapy by rebamipide nanoparticles. Pharmaceutics.

[70] Weng YH, Ma XW, Che J, Li C, Liu J, Chen SZ (2017). Nanomicelle-assisted targeted ocular delivery with enhanced antiinflammatory efficacy in vivo. Adv Sci (Weinh)..

[71] Lee D, Lu Q, Sommerfeld SD, Chan A, Menon NG, Schmidt TA (2017). Targeted delivery of hyaluronic acid to the ocular surface by a polymer-peptide conjugate system for dry eye disease. Acta Biomater.

[72] Lim MJ, Hurst RK, Konynenbelt BJ, Ubels JL (2009). Cytotoxicity testing of multipurpose contact lens solutions using monolayer and stratified cultures of human corneal epithelial cells. Eye Contact Lens.

