# Supplementary material for: Anti-inflammatory and antioxidative effects of gallic acid on experimental dry eye: in vitro and in vivo studies
Source: Eye Vis (Lond). 2023 May 1;10:17. doi: 10.1186/s40662-023-00334-5 (PMC10150500; doi:10.1186/s40662-023-00334-5)

**Additional methods**

- 1. Methods for screening drug concentrations in animal experiments

After preliminary screening to exclude existing ocular surface diseases, the mice were randomized into 5 groups for experimental dry eye (EDE)-induced or eye drop treatment for 5 days. The groupings were as follows: 1) the normal control group (NC), mice that did not receive EDE and were not given eye drops; 2) the EDE group, mice that received EDE but were not given eye drops; 3) the gallic acid (GA, 10 mg/mL) + EDE group, mice that received both EDE and treated with eye drops made from 10 mg/mL GA dissolved in 0.01 M phosphate buffered saline (PBS) eye drops (pH = 7.4); 4) the GA (5 mg/mL) + EDE group, mice that received EDE and treated with eye drops made from 5 mg/mL GA dissolved in 0.01 M PBS eye drops (pH = 7.4); 5) the GA (1 mg/mL) + EDE group, mice that received EDE and treated with eye drops made from 1 mg/mL GA dissolved in 0.01 M PBS eye drops (pH = 7.4). After 5 days, corneal fluorescein sodium staining was performed and mice euthanized by pentobarbital injection.

1.2 The gating strategy for the scatter plot of flow cytometry

Cells were considered ROS-positive if the fluorescence intensity exceeds 5 times (RAW264.7 cells) or 10 times (Human corneal epithelial cells) of the average fluorescence intensity of the control cells (normal cells without any treatment).

**Additional results**

Figure S1. The effect of pre-treatment with gallic acid (GA) on the viability of human corneal epithelial cells (HCECs) and RAW264.7 cells. The effect of GA on the viability of HCECs (**a**) and RAW264.7 cells (**b**). Data are presented as mean ± SD (n = 6). ***P*<0.01, **P*<0.05 compared to the control group (0 μM gallic acid).


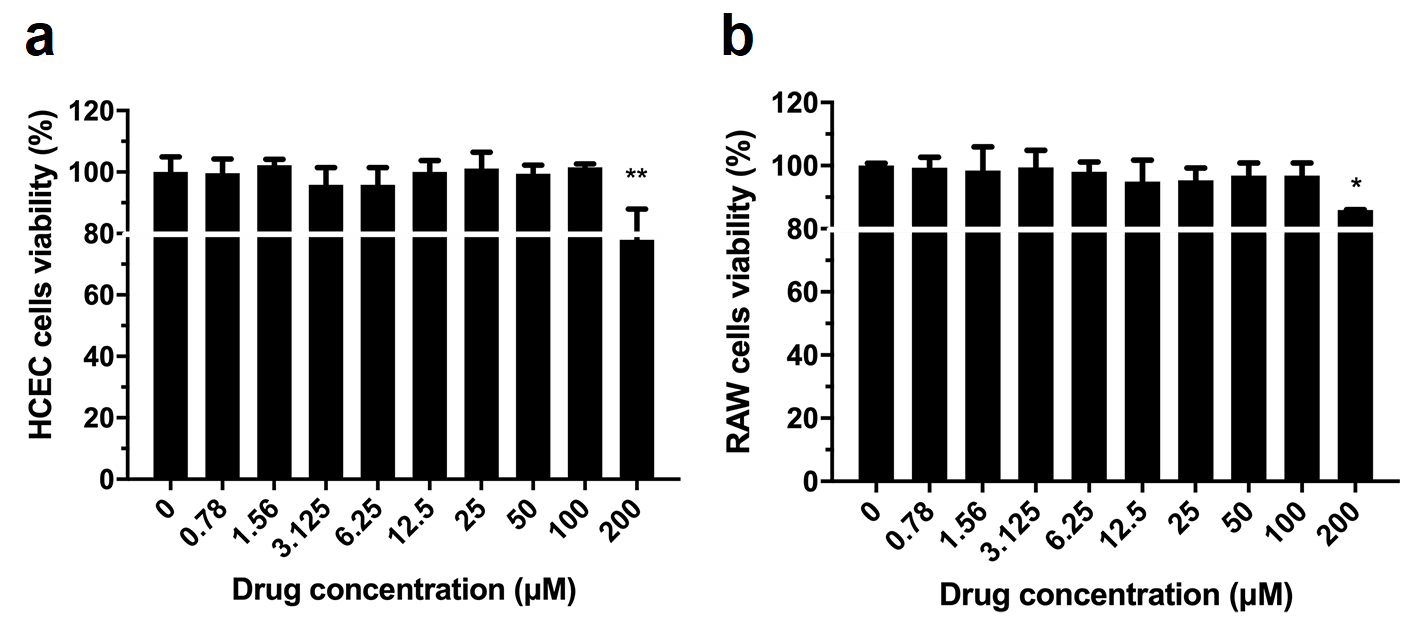


Figure S2. Wound healing results. Control (**a**) and 100 μM gallic acid (**b**) on wound closure was visualized at 0 h post scratch of human corneal epithelial cell (HCEC) monolayer; Control (**c**) and 100 μM gallic acid (**d**) were 24 h after wound healing. **e** The percentage reduction of the average wound width at 6 h, 18 h and 24 h post scratch of HCEC monolayer. Data are presented as mean ± SD (n = 6).


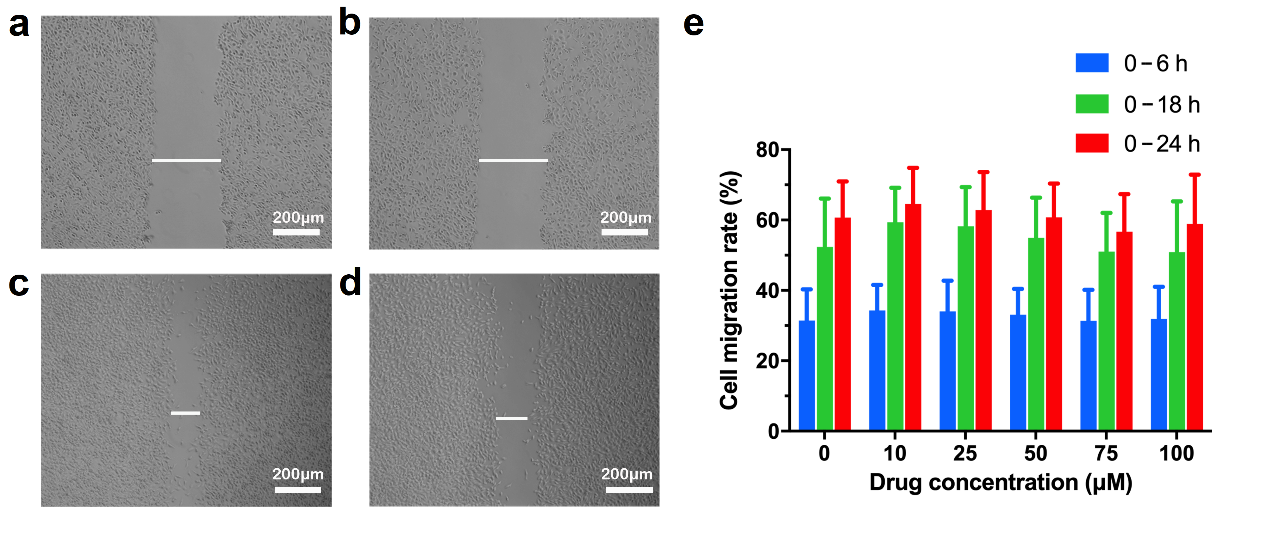


Figure S3. Gallic acid (GA) has good long-term biocompatibility. **a** Treatment plan and observation plan; **b** Ocular surface and conjunctiva images, fluorescein sodium staining of the cornea and hematoxylin and eosin (H&E) staining of the cornea after the eyes were treated with phosphate buffered saline (PBS) solution and gallic acid solution (5 mg/mL) at 10 days post instillation; **c** Draize Test score for ocular surface irritation over 10 days. **d** Intraocular pressure changes over 10 days. Data are presented as mean ± SD (n = 3).


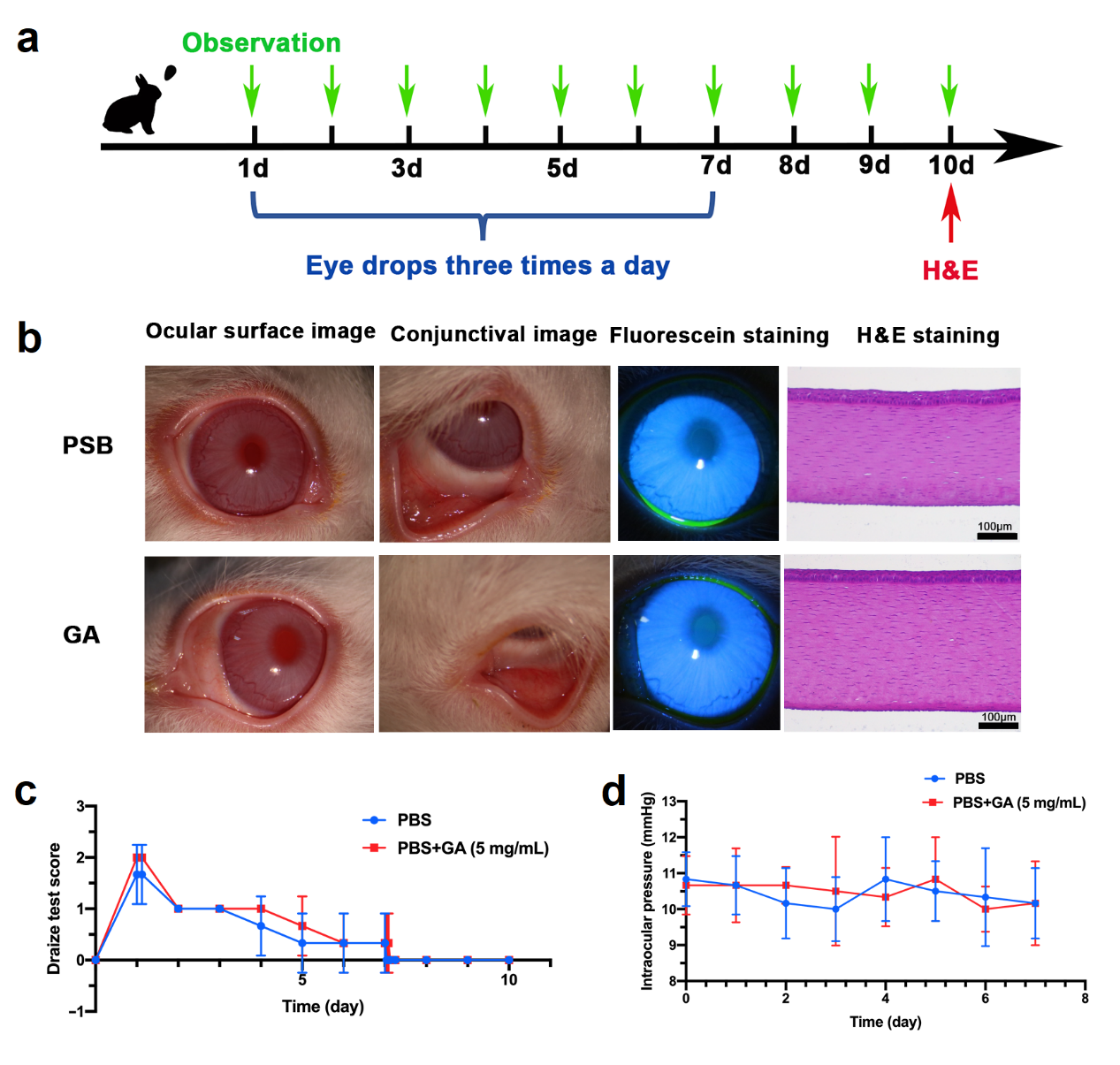


Figure S4. Representative images of the gate plot of intracellular reactive oxygen species (ROS) production in RAW264.7 (**a**) and human corneal epithelial cells (HCECs) (**b**). The production of ROS was measured by ﬂow cytometry using the ﬂuorescent probe 2’,7’-Dichlorodihydrofluorescein diacetate (DCFH-DA). SSC-A, side scatter-area; FITC-A, fluorescein isothiocyanate-area; GA, gallic acid; LPS, lipopolysaccharide


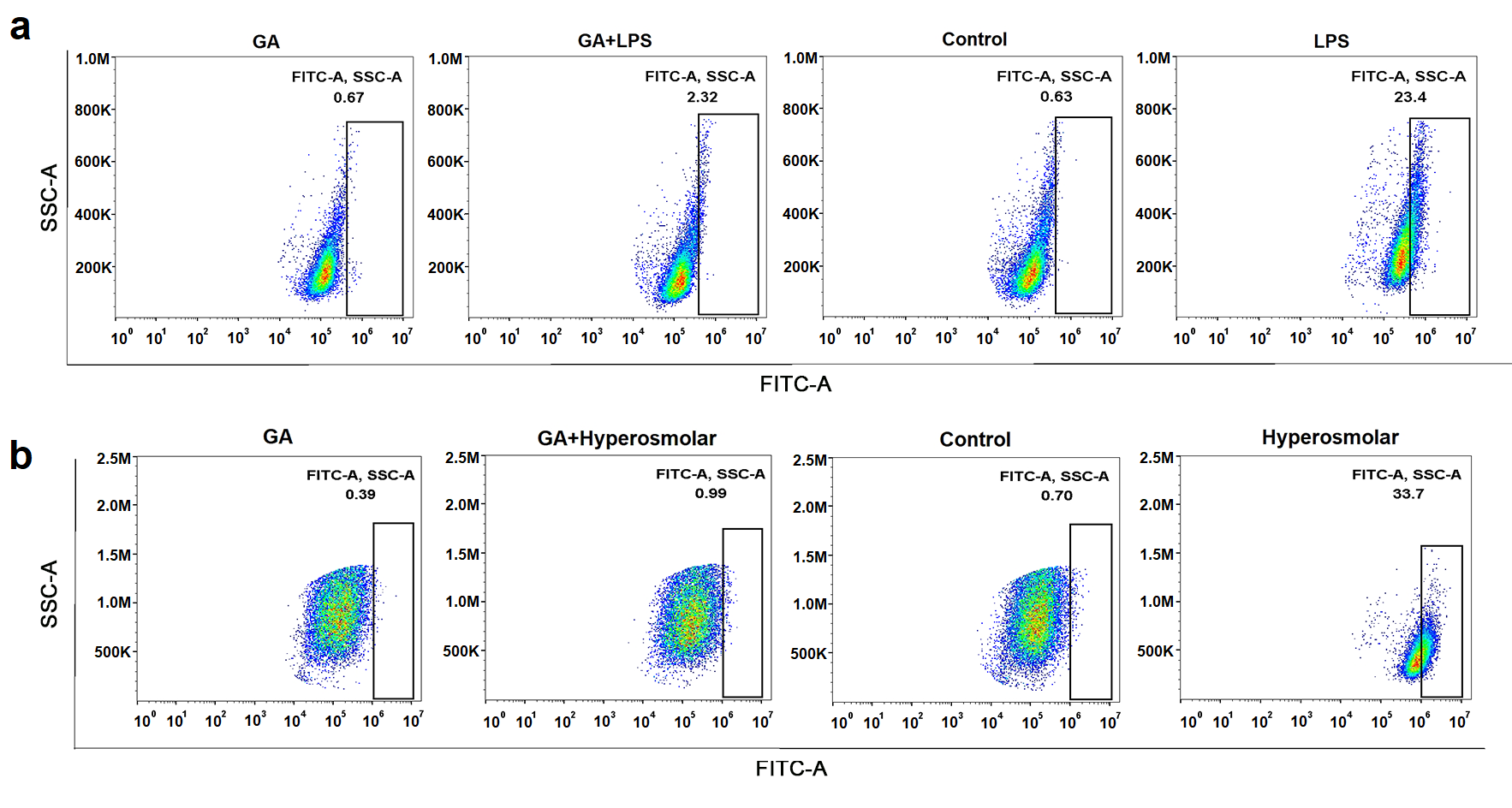


Figure S5. 5 mg/mL and 10 mg/mL gallic acid (GA) can significantly reduce corneal fluorescein sodium spotting. Corneal ﬂuorescein representative ﬁgures (**a**) and staining scores (**b**) show the staining of the NC, EDE, EDE + 10 mg/mL GA, EDE + 5 mg/mL GA, EDE + 1 mg/mL GA groups on the fifth day after desiccant stress. The data are presented as mean ± SD (n = 3). NC, normal control, not received EDE, not given eye drops; EDE, experimental dry eye, received EDE, not given eye drops; EDE + 10 mg/mL GA, received EDE, given 10 mg/mL GA eye drops; EDE + 5 mg/mL GA, received EDE, given 5 mg/mL GA eye drops; EDE + 1 mg/mL GA, received EDE, given 1 mg/mL GA eye drops. *****P*<0.001 compared to the EDE group.


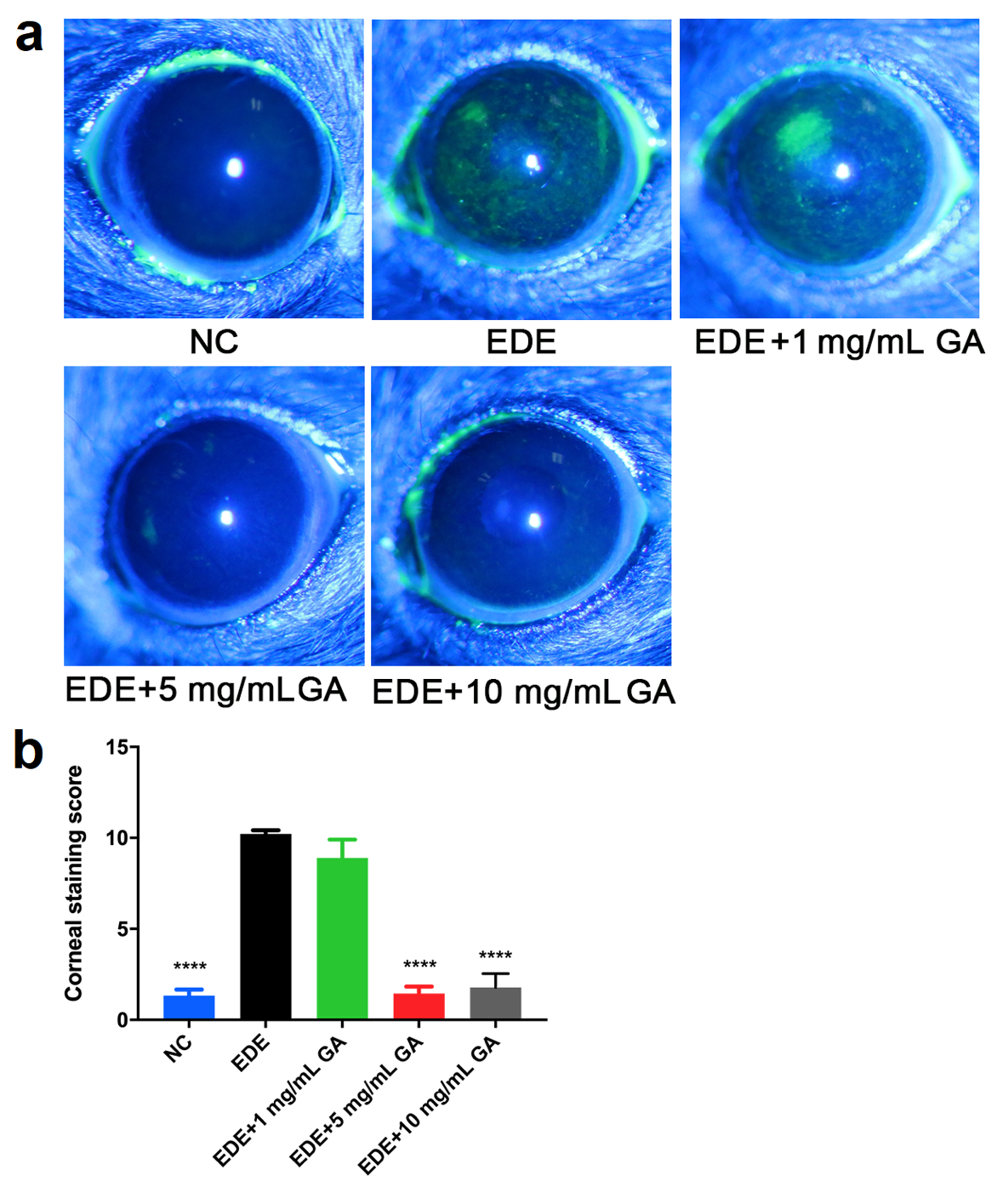


Figure S6. Gallic acid (GA) can prevent corneal fluorescein sodium spot staining and cornea epithelial cell apoptosis. Corneal ﬂuorescein representative ﬁgures (**a**) and staining scores (**b**) show the staining on the fifth day after desiccant stress. Apoptotic corneal epithelial cell count (**c**) and representative ﬁgures (**d**) show the apoptosis conditions 5 days after desiccant stress. The data are presented as mean ± SD (n = 6). NC, normal control; EDE, experimental dry eye; PBS, phosphate buffered saline; P, preventive effect of the drug; P-NC, not received EDE, not given eye drops; P-EDE: received EDE, not given eye drops; P-EDE+PBS: received EDE, given PBS eye drops; P-EDE+GA: received EDE, given GA eye drops. *****P*<0.001 compared to the P-EDE group.


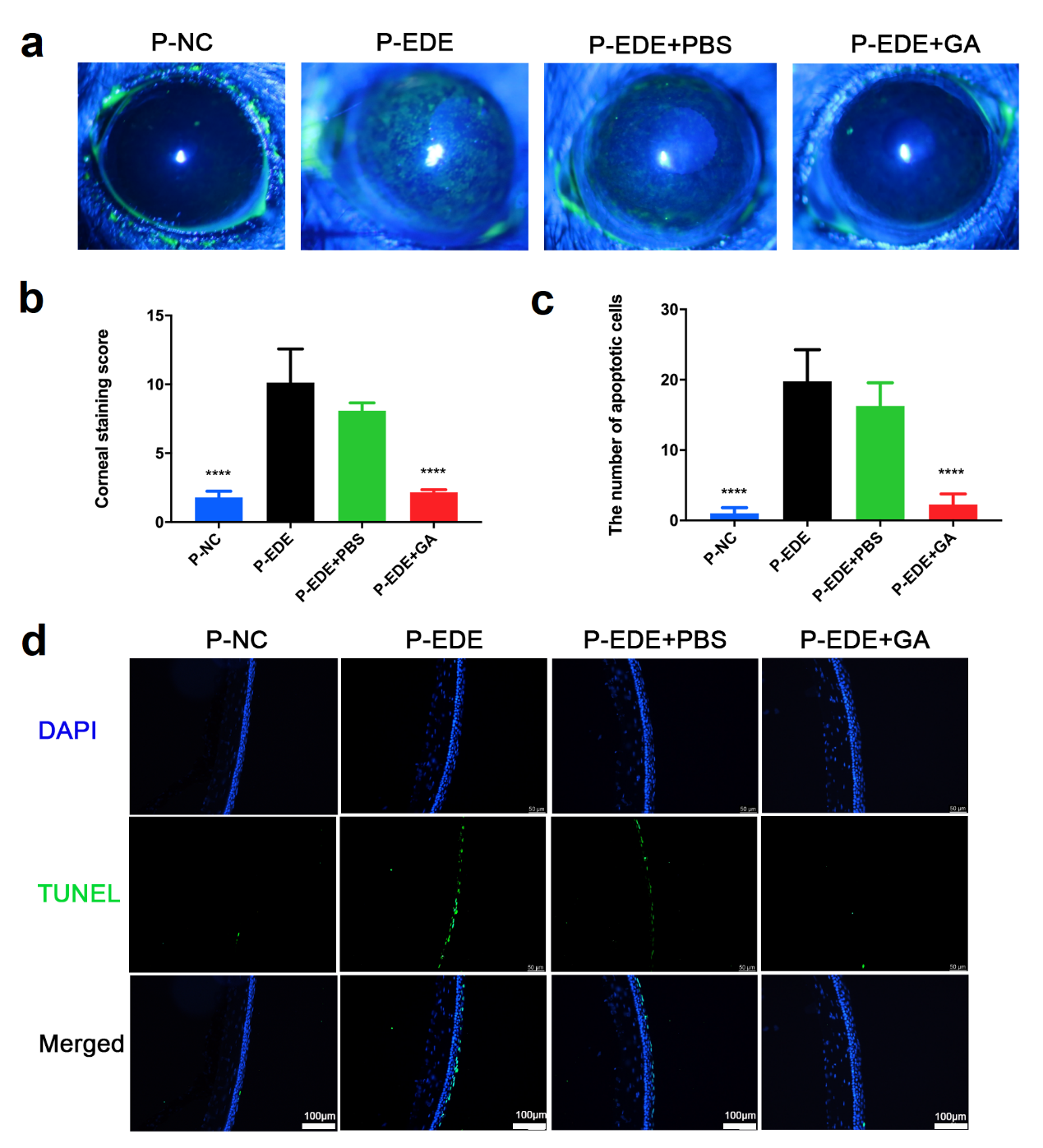


Figure S7. Gallic acid (GA) protects goblet cells. Goblet cells representative ﬁgures (**a**) and goblet cell count (**b**). The data are presented as mean ± SD (n = 3). NC, normal control; EDE, experimental dry eye; PBS, phosphate buffered saline; P: preventive effect of the drug; P-NC: not received EDE, not given eye drops; P-EDE: received EDE, not given eye drops; P-EDE+PBS: received EDE, given PBS eye drops; P-EDE+GA: received EDE, given GA eye drops. ****P*<0.005 compared to the P-EDE group.


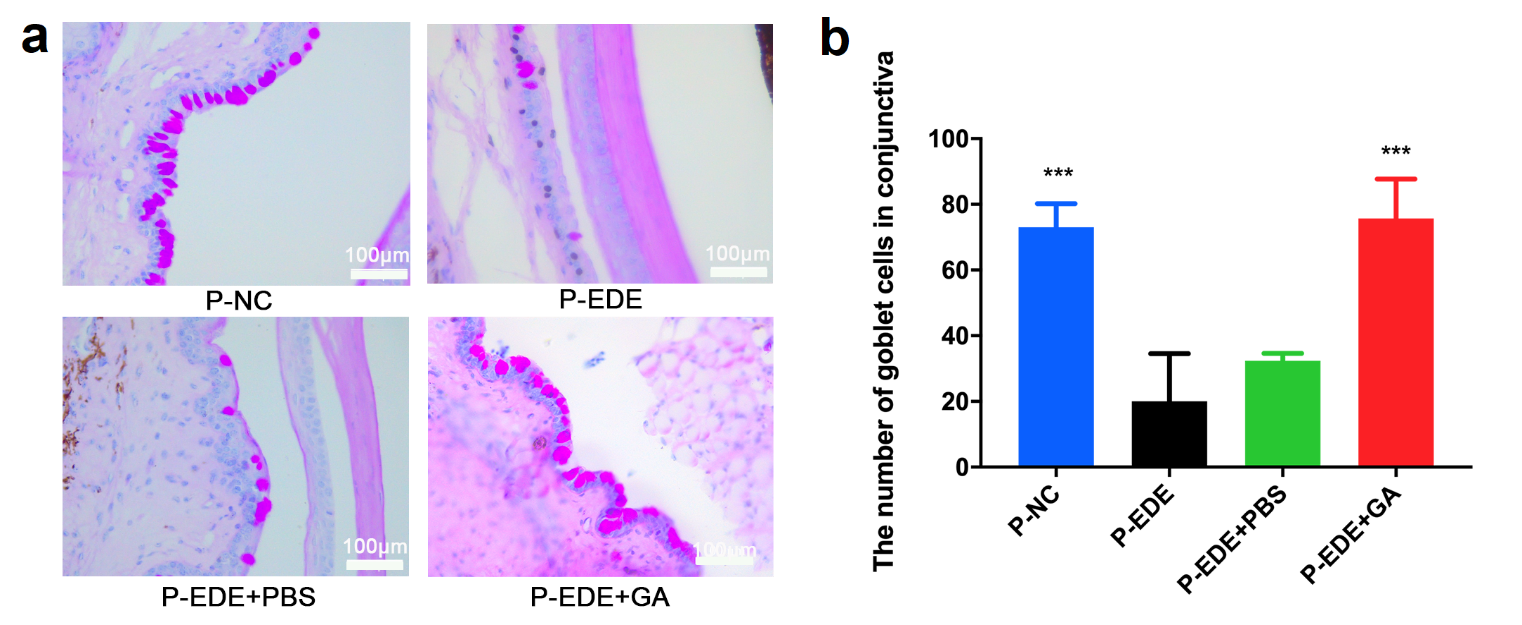


Figure S8. Gallic acid (GA) inhibits the elevation of inflammatory factors in the cornea and conjunctiva. **a, b** respectively show IL-6 and IL-1β levels per mg of the cornea. **c, d** respectively show IL-6 and IL-1β levels per mg of the conjunctiva. The data are presented as mean ± SD (n = 3). NC, normal control; EDE, experimental dry eye; PBS, phosphate buffered saline; P: preventive effect of the drug; P-NC: not received EDE, not given eye drops; P-EDE: received EDE, not given eye drops; P-EDE+PBS: received EDE, given PBS eye drops; P-EDE+GA: received EDE, given GA eye drops. ***P*<0.01, **P*<0.05 compared to the P-EDE group.


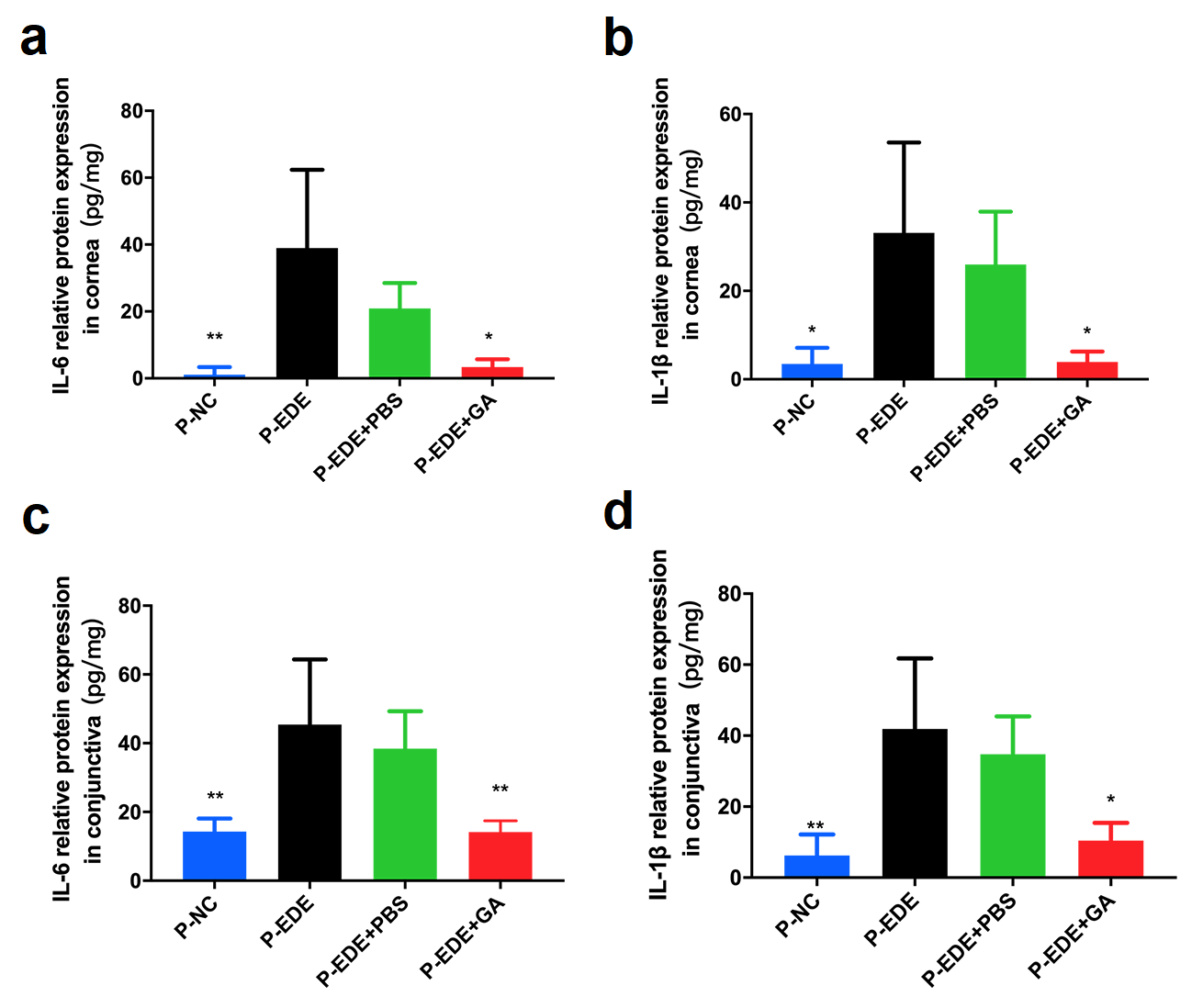

Supplement: Supplementary file 1 — Additional file 1: Figure S1. The effect of pre-treatment with gallic acid (GA) on the viability of human corneal epithelial cells (HCECs) and RAW264.7 cells. The effect of GA on the viability of HCECs (a) and RAW264.7 cells (b). Data are presented as mean ± SD (n = 6). **P < 0.01, *P < 0.05 compared to the control group (0 μM gallic acid). Figure S2. Wound healing results. Control (a) and 100 μM gallic acid (b) on wound closure was visualized at 0 h post scratch of human corneal epithelial cell (HCEC) monolayer; Control (c) and 100 μM gallic acid (d) were 24 h after wound healing. e The percentage reduction of the average wound width at 6 h, 18 h and 24 h post scratch of HCEC monolayer. Data are presented as mean ± SD (n = 6). Figure S3. Gallic acid (GA) has good long-term biocompatibility. a Treatment plan and observation plan; b Ocular surface and conjunctiva images, fluorescein sodium staining of the cornea and hematoxylin and eosin (H&E) staining of the cornea after the eyes were treated with phosphate buffered saline (PBS) solution and gallic acid solution (5 mg/mL) at 10 days post instillation; c Draize Test score for ocular surface irritation over 10 days. d Intraocular pressure changes over 10 days. Data are presented as mean ± SD (n = 3). Figure S4. Representative images of the gate plot of intracellular reactive oxygen species (ROS) production in RAW264.7 (a) and human corneal epithelial cells (HCECs) (b). The production of ROS was measured by flow cytometry using the fluorescent probe 2’,7’-Dichlorodihydrofluorescein diacetate (DCFH-DA). SSC-A, side scatter-area; FITC-A, fluorescein isothiocyanate-area; GA, gallic acid; LPS, lipopolysaccharide. Figure S5. 5 mg/mL and 10 mg/mL gallic acid (GA) can significantly reduce corneal fluorescein sodium spotting. Corneal fluorescein representative figures (a) and staining scores (b) show the staining of the NC, EDE, EDE + 10 mg/mL GA, EDE + 5 mg/mL GA, EDE + 1 mg/mL GA groups on the fifth day after desiccant stres [file 40662_2023_334_MOESM1_ESM.docx]
